# A Systems-Based Analysis of *Plasmodium vivax* Lifecycle Transcription from Human to Mosquito

**DOI:** 10.1371/journal.pntd.0000653

**Published:** 2010-04-06

**Authors:** Scott J. Westenberger, Colleen M. McClean, Rana Chattopadhyay, Neekesh V. Dharia, Jane M. Carlton, John W. Barnwell, William E. Collins, Stephen L. Hoffman, Yingyao Zhou, Joseph M. Vinetz, Elizabeth A. Winzeler

**Affiliations:** 1 Department of Cell Biology ICND 202, The Scripps Research Institute, La Jolla, California, United States of America; 2 Division of Infectious Diseases, Department of Medicine, University of California San Diego School of Medicine, La Jolla, California, United States of America; 3 Sanaria Inc., Rockville, Maryland, United States of America; 4 Department of Medical Parasitology, New York University Langone Medical Center, New York, New York, United States of America; 5 Centers for Disease Control and Prevention, Division of Parasitic Diseases, Atlanta, Georgia, United States of America; 6 Genomics Institute of the Novartis Research Foundation, San Diego, California, United States of America; Johns Hopkins Bloomberg School of Public Health, United States of America

## Abstract

**Background:**

Up to 40% of the world's population is at risk for *Plasmodium vivax* malaria, a disease that imposes a major public health and economic burden on endemic countries. Because *P. vivax* produces latent liver forms, eradication of *P. vivax* malaria is more challenging than it is for *P. falciparum*. Genetic analysis of *P. vivax* is exceptionally difficult due to limitations of *in vitro* culture. To overcome the barriers to traditional molecular biology in *P. vivax*, we examined parasite transcriptional changes in samples from infected patients and mosquitoes in order to characterize gene function, define regulatory sequences and reveal new potential vaccine candidate genes.

**Principal Findings:**

We observed dramatic changes in transcript levels for various genes at different lifecycle stages, indicating that development is partially regulated through modulation of mRNA levels. Our data show that genes involved in common biological processes or molecular machinery are co-expressed. We identified DNA sequence motifs upstream of co-expressed genes that are conserved across *Plasmodium* species that are likely binding sites of proteins that regulate stage-specific transcription. Despite their capacity to form hypnozoites we found that *P. vivax* sporozoites show stage-specific expression of the same genes needed for hepatocyte invasion and liver stage development in other *Plasmodium* species. We show that many of the predicted exported proteins and members of multigene families show highly coordinated transcription as well.

**Conclusions:**

We conclude that high-quality gene expression data can be readily obtained directly from patient samples and that many of the same uncharacterized genes that are upregulated in different *P. vivax* lifecycle stages are also upregulated in similar stages in other *Plasmodium* species. We also provide numerous examples of how systems biology is a powerful method for determining the likely function of genes in pathogens that are neglected due to experimental intractability.

## Author Summary

Most of the 250 million malaria cases outside of Africa are caused by the parasite *Plasmodium vivax*. Although drugs can be used to treat *P. vivax* malaria, drug resistance is spreading and there is no available vaccine. Because this species cannot be readily grown in the laboratory there are added challenges to understanding the function of the many hypothetical genes in the genome. We isolated transcriptional messages from parasites growing in human blood and in mosquitoes, labeled the messages and measured how their levels for different parasite growth conditions. The data for 5,419 parasite genes shows extensive changes as the parasite moves between human and mosquito and reveals highly expressed genes whose proteins might represent new therapeutic targets for experimental vaccines. We discover sets of genes that are likely to play a role in the earliest stages of hepatocyte infection. We find intriguing differences in the expression patterns of different blood stage parasites that may be related to host-response status.

## Introduction

Renewed efforts to combat malaria have focused on the goal of total eradication. While most attention is on the more deadly *Plasmodium falciparum* malaria, *Plasmodium vivax* is the most geographically widespread human malaria parasite causing an estimated 80–250 million cases of vivax malaria each year [1]. *P. vivax* malaria has traditionally been found outside of tropical areas and was endemic throughout North America and Europe until the introduction of DDT. Despite the large burden of disease caused by *P. vivax*, it is overlooked and left in the shadow of the enormous public health burden caused by *P. falciparum* in sub-Saharan Africa. The widely held misperception of *P. vivax* as being relatively infrequent, benign, and easily treated explains it's nearly complete neglected across the range of biological and clinical research. In fact, *P. vivax* malaria seriously threatens more people than has been historically appreciated. Recent reports [2]–[4] provide abundant evidence that challenge the paradigm that *P. vivax* infection causes benign disease*: P. vivax* malaria may result in severe symptoms similar to *P. falciparum*. Selective pressure for resistance to malaria has had a great influence on the human genome and most Africans are immune to *P. vivax* malaria due to mutations in the Duffy receptor that the parasites use to invade the red cell.

A fundamental difference between *P. vivax* and *P. falciparum* is the formation of dormant liver stage parasites called hypnozoites that are resistant to schizonticidal drugs that kill erythrocytic stage parasites. Despite schizonticidal drug therapy, the patient may experience multiple relapses months or years following the primary infection. Attempts to eradicate malaria will depend on having effective and non-toxic drugs that target *P. vivax* liver stage hypnozoites. Hypnozoite biology is poorly understood but is likely related to persistence of the parasite in locales where mosquito populations vary seasonally. Little is known about what triggers a relapse but some strains form different numbers of hypnozoites and have different relapse frequencies, which may be correlated with latitude[5]. There is controversy about whether hypnozoites represent a different lifecycle stage or merely represent an arrested early exo-erythrocytic phase. Almost nothing is known about metabolic activity in the hypnozoite, thwarting all efforts at rationale drug design. The mechanism of the only drug known to eradicate hypnozoites, the 8-aminoquinoline drug, primaquine, is still not completely understood.

While rodent models have furthered our understanding of *Plasmodium* liver stage development, they do not form hypnozoites. The only other available model for studying hypnozoite biology is the closely related *P. cynomolgi* species that must be studied in the rhesus macaque. Studies of *P. vivax* biology still depend on obtaining erythrocyte stage parasites or sporozoites from infected humans or non-human primates or their mosquito vectors, respectively. Drug sensitivity testing remains difficult. In an earlier era compounds were tested for activity against hypnozoites in prisoners who had been given malaria [6]. Drug testing *in vitro* is limited by current difficulties in culture techniques. Only a few genetic manipulations of *P. vivax* have been successful [7]. These impediments have discouraged many researchers from working on *P. vivax*, and limited the potential for molecular genetic analysis of this elusive liver stage.

Given that forward and reverse genetic methods, which have been powerful in *P. falciparum*, are not readily available for investigating the genome of *P. vivax*, comparative genomics and virtual genetic methods offer the best opportunities to elucidate *P. vivax* gene function. Previous gene expression profiling of *P. falciparum* [8], [9] *P. yoelii* [10] throughout many developmental life cycle stages in the mammalian host and insect vector, and in *P. vivax* blood stages [11], [12] has provided fundamental insight into *Plasmodium* biology and illustrated how gene function can be predicted based on gene expression patterns throughout development. The power of this method is to assure that there is sufficient diversity in different stage parasites to allow for the delineation of distinct patterns of gene expression.

Here we used a systems biology approach to characterize the *P. vivax* transcriptome. Using a custom high-density tiling microarray, we obtained a diverse set of gene expression data from human and mosquito stages including sporozoites, gametes, zygotes and ookinetes, and *in vivo* asexual blood stages obtained from infected patients in the Peruvian Amazon. These data are combined with published short term *in vitro* culture data [11]. Using a guilt-by-association approach we create hypotheses about the function of many uncharacterized genes. Comparison to datasets of *P. falciparum* and *P. yoelii* reveals conserved and species-specific patterns. This analysis provides new insights into the metabolic state of parasites growing within humans. It shows that many of the orthologs of *P. falciparum* transcripts needed for exo-erythrocytic development are present in the *P. vivax* sporozoites suggesting that slight modifications in exo-erythrocytic development may allow hypnozoite formation.

## Materials and Methods

### Ethics statement

The protocol used to collect human blood samples for this work was approved by the Human Subjects Protection Program of The Scripps Research Institute, and the University of California, San Diego and by the Ethical Committees of Universidad Peruana Cayetano Heredia and Asociacion Benefica PRISMA, Iquitos, Peru. Written informed consent was obtained from each subject or the parent, in the case of minors. The consent form states in English and Spanish that samples may be used for any scientific purpose involving this or any other project, now or in the future and that the samples may be shared with other researchers.

### Collection of patient samples and preparation of parasite cells

Patients who presented to local health clinics in the Peruvian Amazon region of Iquitos with typical signs and symptoms of malaria were evaluated by light microscopy examination of Giemsa-stained blood smears to have *P. vivax* parasitemia. After informed consent, 20 ml of blood was drawn into heparinized vaccutainer tubes, placed in a portable incubator maintained between 37 and 39°C, and transported to laboratory facilities within 30 minutes. Blood was centrifuged at 900×g for 5 minutes and plasma was removed. Red cells were resuspended in 2 volumes of suspended animation (SA) solution (10 mM Tris, 170 mM NaCl, and 10 mM glucose pH 7.4) and passed through a cellulose column (CF-11 powder, Whatman Ltd) placed in a 37°C incubator, to remove white blood cells. Asexual parasites were enriched by gradient purification. Following filtration, cells were washed twice in SA solution at 900 g for 5 minutes and then resuspended in a 1∶3 v/v ratio in SA solution. For the production of gametes/zygotes and ookinetes in vitro, The red cells containing parasites were resuspended in exflagellation medium (10 mM Tris, 170 mM NaCl, 10 mM glucose, 25 mM NaCO3, 10% AB+ human serum, 50 mM xanthurenic acid) to induce parasite emergence from gametocytes, exflagellation and fertilization. The cell suspension was then layered over a discontinuous gradient of 38%, 42% and 50% Percoll (Sigma, USA) in RPMI 1640 medium (Invitrogen USA) and centrifuged at 600 g for 15 minutes. Female gametocytes at the 38–42% interface were removed from the gradient. Red cells containing asexual parasites which sediment below the gradient were collected and washed separately in SA solution at 900 g for 10 minutes. Zygotes and ookinetes and untransformed gametes at the 11%–16% interface were collected, washed in PBS and resuspended in Trizol and stored at −70°C. Microscopic analysis of cellular morphology confirmed the enrichment of sexual stages. Small aliquots of purified cells were stained and 60 fields were examined to determine the relative percentage of asexual and gametocyte cells (Table 1). Red cells from asexual enrichments were subjected to lysis by a 0.1% Saponin solution in PBS for 15 minutes at 4°C. Parasites were pelleted at 1200 g for 5 minutes and washed three times in PBS before resuspension in Trizol and storage at −70°C for shipment to the Scripps Research Institute.

**Table 1:** *P. vivax* blood sample asexual profile and stage specificity.

Asexual Sample	Asexual Group	Rings and Early Trophs	Schizonts	Gametocytes	% Asexuals	% Gametocytes
CM008	1	169	0	0	100%	0%
CM101	1	240	0	19	93%	7%
CM108	1	292	0	36	89%	11%
CM109	1	143	0	22	87%	13%
CM114	1	316	0	20	94%	6%
CM115	Unique	82	0	20	80%	20%
CM012	2	148	0	88	63%	37%
CM013	2	291	0	36	89%	11%
CM008 Filtered	1	108	0	0	100%	0%
CM101 Filtered	1	73	0	8	90%	10%
CM108 Filtered	1	201	0	7	97%	3%
CM109 Filtered	1	112	0	6	95%	5%
CM114 Filtered	1	332	0	14	96%	4%
CM115 Filtered	Unique	101	0	17	86%	14%
CM012 Filtered	2	139	0	19	88%	12%
CM013 Filtered	2	37	0	1	97%	3%
Sexual Stage	Gametes	Total %	Zygotes	Total %	Ookinetes	Total %
Gamete/Zygote	101	80%	26	20%	0	0%
Ookinete	0	0%	7	9%	68	91%

Parasite cell stages present in each patient blood sample at time of collection are listed first. Numbers of cell stages were based on counting microscope 100X objective view fields until researchers observed a total of 200 white blood cells. Filtered sample numbers indicate the parasite cell stages present in 20 fields in each sample following filtration to remove white blood cells and Percoll gradient centrifugation to enrich for asexual stages. All asexual samples contained rings and early trophozoite stages, with no late trophozoites or schizonts. Percoll gradient centrifugation reduced gametocytes in all asexual samples, but a small percentage remained in the sample from which RNA was prepared. Sexual stage sample data indicate parasite cell stages following *in vitro* cultivation present in 30 fields at the time of RNA sample collection. All samples were collected between November 2007 and July 2008 and were not checked for clonality.

### *P. vivax* whole genome-tiling microarray design

We designed a Affymetrix custom *P. vivax* whole-genome tiling microarray with 4.2 million 25-bp probes covering both strands at six base pair spacing, based on the genome assembly of 2809 contigs (PlasmoDB Ver. 5.4). This microarray includes 1.7 and 2.3 million probes uniquely mapped to coding regions and non-coding regions, respectively. Altogether 5419 *P. vivax* genes are represented on the array, 4676 of which have *P. falciparum* orthologs. This array will be made available for purchase from Affymetrix, part number PvivaxLi520507.

### RNA preparation and microarray hybridization

RNA was purified by Chloroform extraction and isopropanol precipitation and purified by RNeasy Mini Kit (Qiagen) following the manufacturer's instructions. RNA was quantified by spectrophotometer and qualitatively analyzed by BioRad Experion RNA StdSens Analysis kit (BioRad). 1 ug of total RNA from asexual blood stage parasites was used to produce cDNA in the Two-cycle cDNA synthesis kit (Affymetrix) and amplified to produce labeled cRNA in the IVT Labeling kit (Affymetrix), and purified using the Genechip Sample Cleanup Module (Affymetrix), according to manufacturer's instructions. For the sporozoite sample, 100 ng of total RNA was used to produce cDNA in the Two-cycle cDNA synthesis kit (Affymetrix) and amplified to produce labeled cRNA in the IVT T7 MEGAScript kit (Affymetrix). Following the first IVT reaction, the cRNA was split into two reactions, containing approximately 500 ng each for the second round of cDNA synthesis and amplified to produce labeled cRNA in the IVT Labeling kit (Affymetrix), and purified using the Genechip Sample Cleanup Module (Affymetrix), according to manufacturer's instructions. 20 ug of amplified cRNA was hybridized to the *P. vivax* tiling microarray for 14 hours. The genechips were washed on Affymetrix Wash Station using standard Affymetrix protocol FlexGE-WS450_00001 and scanned on the Affymetrix scanner. The Affymetrix CEL files microarray data are available for download from our companion website (http://carrier.gnf.org/publications/Pv). Gene expression data are also visible in PlasmoDB on the *P. vivax* gene info pages. For more information on gene expression interpretation from our custom whole genome tiling array please see the website.

### Differential gene expression and OPI analysis

All gene expression values were subjected to a probe-level two-way ANOVA test to determine variability of expression across all samples [13]. A total of 4,326 genes were identified as differentially expressed with p_ANOVA_ <0.05 and FC >2 (Table S1) and were subjected to further OPI clustering analysis. All data can be downloaded from the companion web site.

All Plasmodium gene descriptions and name aliases were downloaded from PlasmoDB (version 6.1). Function annotation data for *P. falciparum*, *P. yoelii*, *P. vivax*, *P. berghei*, *P. chaubaudi* and *P. knowlesi* were obtained from PlasmoDB, then merged with *P. falciparum* function annotation data from Gene Ontology (November 2009 release). We had previously collected gene co-citation data through Google Scholar and NCBI [10], this dataset is appended with co-citation data found in “gene notes” section of the PlasmoDB annotation files. We also considered previously published data such as *P. falciparum* protein complexes [14], *P. falciparum* cell cycle *k*-means clusters [9], and our own literature-based annotations [10]. For each gene list, we further recruited additional gene members that are homologs or orthologs among *Plasmodium* species according to the latest OrthoMCL database (version 3). The final annotation database contains 3,732 gene lists that contains at least one *P. vivax* gene, including 2,538 GO groups, 1,066 literatures, 84 custom GNF lists, 29 complexes and 15 cell cycle clusters. From these 2,002 lists contains at least two differentially expressed *P. vivax* genes, therefore were used to create clusters of co-regulated genes that share gene ontology annotation using the ontology-based pattern identification (OPI) algorithm described previously [15]-[17]. To weight samples correctly, replicate samples were weighted 50% each and effected counted as one sample. When our dataset were merged with Bodzech data, the overall weighting of samples in each data set was adjusted so that two data sets contributed equally in the clustering analysis.

**Figure 1:**
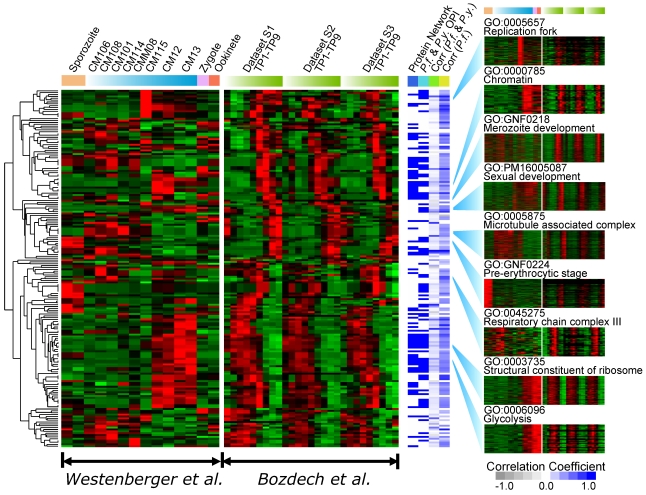
Gene expression patterns of 192 statistically significant cellular function clusters across all 41 *P. vivax* samples. Each heat map row represents the common expression pattern shared by its cluster members and these profiles are hierarchically clustered so transcriptionally-related cellular functions are in close vicinity. For nine selected clusters with distinct life cycle expression profiles, the detailed gene-by-gene expression heat maps are shown to illustrate the high correlation within any given cluster. Additional evidences collected for each OPI cluster is summarized in a white-blue heat map. Blue color indicates a favorable piece of evidence, *i.e.*, either the cluster members form statistically significant protein networks, or the OPI cluster was also found based on previous analyses and reanalyses of the published 54-sample *P. falciparum* and *P. yoelii* life cycle data set [10]. The average Pearson correlation coefficient of the expression profiles of *P. falciparum* orthologs of the cluster members are also color coded, so that blue represents a high positive correlation and gray represents a low negative correlation.

Visualization of the OPI expression patterns in Figure 1 used the OPI query pattern data as described previously [17]. Briefly, the query pattern is the best representative expression pattern of a given cluster, and was typically derived from common pattern shared by most of the known GO members of the cluster. Each query profile is normalized by subtracting the mean and divided by the standard deviation, a standard practice used for heat map plotting. The query patterns representing the 192 gene functions were hierarchically clustered so that related biological processes are close to each other.

### Cross validation with published gene expression datasets

For each of the 192 statistically significant *P. vivax* OPI clusters, we identified the *P. falciparum* and *P. yoelii* orthologs of its cluster members. We then calculated the average pair-wise Pearson correlation coefficient of the *P. falciparum* orthologs in a previously published cell cycle dataset of 2,235 genes. The correlation coefficients are almost all positive with a median at 0.45. We also calculated using a similar approach the average correlation coefficients of the orthologs in a combined *P. yoelii* and P. *falciparum* life cycle dataset. All results are available in Table S2.

For each piece of *P. vivax* gene function prediction, we checked if its *P. falciparum* or *P. yoelii* orthologs were also predicted to be in the same function category based on the previous *P. yoelii* and P. *falciparum* life cycle dataset. Both previously published OPI clusters [10] and new OPI clusters based on the latest gene annotation database were used in this cross validation analyses. Notice here an orthlog was considered to be associated with a GO group as long as it appeared in the group or one of its descendant groups. Support was found for 89 of the 192 OPI clusters.

### Cross validation with published yeast two-hybrid and literature datasets

We first projected the previously published *P. falciparum* yeast two-hybrid protein interaction database into *P. vivax* by introducing interactions to all the corresponding *P. vivax* ortholog pairs using OrthoMCL version 3. We then constructed a protein network for each OPI cluster and recorded the size of the network. To evaluate the statistical significance of the resultant network, we replaced the cluster members through random sampling and repeated the network construction process 1000 times. The p-value of the network was estimated based on the probability of an equal or more complex network to occur by chance. The whole process was carried out by either only considering direct protein-protein interactions or including indirect protein-protein interactions and whichever led to a better p-value was selected for presentation. An indirect protein-protein interaction refers to two proteins interact via another protein. A total of 63 networks were determined to have p-values <0.05.

It was previously shown that genes co-cited in a literature tend to be more correlated in their expression profile compared to gene members of the same GO group, probably due to the careful review process involved in the publication [10]. Therefore, it could be worthwhile introducing virtual protein-protein interactions among genes co-cited. With these virtual interactions appended to the yeast two-hybrid dataset, we repeated the above network evaluation processes for non-literature derived OPI clusters and identified 11 additional networks.

Protein networks were visualized using Cytoscape (Version 6.1), where nodes can be color coded according to the level of confidence in their function predictions, and edges can be color coded to reflect the data sources.

### Motif analysis

Statistical enrichment of motifs in upstream regions was determined using GeneSpring 7.3 software, using the “search for regulatory sequences” with parameters of 0 to 1000 bases, motif sizes of 5 to 9 bps and allowing for up to two N's in the central region. Only cutoffs with a corrected p-value of less than 0.05 are reported except for very small clusters.

### Preparation and analysis of *P. yoelii* and *P. falciparum* sporozoite RNA

*P. vivax* sporozoites were obtained from Sanaria, Inc. from mosquitoes fed on *P. vivax* infected chimpanzees infected with India VII strain *P. vivax* [18]. Sporozoites were dissected from mosquito salivary glands and purified. Approximately 150,000 sporozoite cells were used for RNA preparation. Similarly purified sporozoites of *P. falciparum* strain 3D7 isolated from mosquitoes infected with *in vitro* cultured parasites were also obtained from Sanaria, Inc.

*P. falciparum* salivary gland sporozoites were obtained from Sanaria, Inc. *P. falciparum* sporozoite RNA was isolated and amplified using Affymetrix kits as described for *P. vivax* sporozoite samples. *P. falciparum* 3D7 strain RNA from *in vitro* synchronized trophozoite stage parasites was isolated and amplified as described for *P. vivax* samples. Amplified cRNA was hybridized to the Pftiling array described previously [19].

To validate the expression comparison of genes that are differentially expressed in sporozoites of *P. vivax* and *P. falciparum*, we performed quantitative reverse transcriptase polymerase chain reaction (qRT-PCR) on 22 genes with orthologs in both species, and two *P. vivax* specific genes. Primers used are listed below. All primer sets were optimized using genomic DNA from 3D7 strain *P. falciparum* and Salvador I strain *P. vivax* at three dilutions of 10 ng/ul, 1 ng/ul and 0.1 ng/ul to ensure that the amplification threshold values accurately reflected the difference in DNA template concentration. Primer sets for the two different species produced similar threshold Ct values (what does Ct stands for) (+/− 1.5 Ct) for all primer sets for both species. An additional aliquot of 150,000 sporozoites for both *P. falciparum* and *P. vivax* from Sanaria, Inc, were used to isolate total RNA using Trizol as described previously. This total RNA sample was split into equally into three reactions to produce single stranded cDNA using reverse transcriptase and a T7-Oligo dT primer from the cDNA synthesis kit (Affymetrix) according to manufacturer's instructions. The single stranded cDNA was used as template for QRT-PCR reactions using the primer sets presented. To account for variability in the input cDNA between different cDNA reactions from the same species, we normalized the threshold Ct values by the average difference between reactions across all genes. We cannot know the identity of any one gene, which is expressed at the exact same level in both species that can be used to control for variation. However, when comparing *P. vivax* and *P. falciparum* threshold Ct values, we found that the highest expressed gene (CSP) and lowest expressed gene (Pv117045 zinc finger) in both species showed very similar threshold Ct values within 1.5 Ct cycles, which was the within the error observed by DNA optimization. Our gene expression data from multiples species, including P. yoelii, as well as proteomic data and conventional experiments show CSP is often one of the most abundant proteins in plasmodium sporozoites. It seems safe to use this in normalization. qRT-PCR reactions were prepared using SYBR GREEN PCR Master Mix (Applied Biosystems) according to manufacturer's instructions, and were run on Applied Biosystems TaqMan machine using SDS 2.2.1 software. Threshold Ct values were determined using default settings and automatic threshold determination. All amplification results were manually inspected to ensure that threshold levels were determined within the logarithmic amplification phase of the reaction for accurate determination of Ct values. Fold difference between *P. falciparum* and *P. vivax* qRT-PCR determined expression values are equal to 2 raised to the power of the difference in Ct values between the two species. qRT-PCR results and primers used are listed in Supplemental Table S2.

The list of *P. vivax* sporozoite-specific genes used to seed the OPI cluster includes: S13, MAC/Perforin (PVX_000810, PFD0430c); SIAP-1 (PVX_000815, PFD0425w); pf52 protein (PVX_001015, PVX_001020, PFD0215c); ECP1, cysteine protease (PVX_003790, PFB0325c); asparagine-rich antigen Pfa35-2 (PVX_081485, PFA0280w); S24, hypothetical protein (PVX_081555, PFA0205w); TRSP (PVX_081560, PFA0200w); TRAP (PVX_082735, PF13_0201); S14, hypothetical protein (PVX_084410, PFL0370w); S25, kinesin-related protein (PVX_084580, PFL0545w); MAEBL (PVX_092975, PF11_0486); S1, hypothetical protein (PVX_094625, PF10_0083); kinesin-related protein (PVX_094710, PFL0545w); conserved hypothetical protein (PVX_097795, PFE0230w); hypothetical protein (PVX_118360, PF14_0404); circumsporozoite (CS) protein (PVX_119355, PFC0210c); early transcribed membrane protein 13, ETRAMP13 (PVX_121950, PF13_0012); S23, conserved hypothetical protein (PVX_123155, PF08_0088); S4, conserved hypothetical protein (PVX_123510, PFL0800c); conserved hypothetical protein (PVX_123750, PFL1075w).

### Discovery of un-annotated gene expression in *P. vivax*

To infer gene expression of un-annotated genes in *P. vivax*, we performed a BLAST search of all *P. falciparum* and *P. knowlesi* annotated genes against the *P. vivax* genome to identify all putative orthologous genes that may not be annotated in *P. vivax*. The BLAST similarity coordinates were used to define the coding region in *P. vivax*. We have not validated the coding sequence for proper gene translation nor have we defined intron-exon boundaries for these genes. These gene boundary definitions were used to pick probes to evaluate the level of gene expression from these regions in the same way as all other annotated *P. vivax* genes. These genes were originally named using the GeneID numbers of their *P. falciparum* and *P. knowlesi* orthologs. We have included these gene expression values for these putative genes in Table S1. We also performed an analysis of all *P. vivax* RNA microarray hybridization data to identify highly transcribed regions of 50 bp that do not overlap with existing gene annotations. We found a few of these regions, but they appeared to correspond to additional exons, intronic regions, or 5′ or 3′ untranslated regions of existing genes. One additional gene identified by this method is the Pv_PF11_0140 gene. We provide putative *P. vivax* Gene ID numbers for these genes based on their position relative to existing flanking genes. We provide a list of these new putative gene coordinates in Supplemental Table S3.

## Results

### Sample collection and microarray analysis

Blood samples were collected from eight different *P. vivax-*infected patients with uncomplicated malaria in Iquitos, Peru. Human leukocytes were removed by filtration and *P. vivax* gametocytes were separated from asexual stage parasites by gradient centrifugation. There was only a small proportion of gametocytes (0–15%) in the final sample (Table 1), so we hereafter refer to these samples as asexual profiles. By microscopy the asexual cells in the patient blood samples appeared to be rings and early trophozoite stages, with no late trophozoite or schizont stages, likely as the result of natural synchronization in the Peruvian patients. For one sample, we put the isolated gametocyte stages into *in vitro* culture and induced sexual stage development to obtain a mixed gamete/zygote stage sample and a mostly pure ookinete stage sample from the patient isolate (Table 1). Additionally, we isolated salivary gland sporozoites from dissected mosquitoes fed on an experimentally infected chimpanzee.

We assayed gene expression using a custom *P. vivax* whole genome tiling microarray with 4.2 million 25-base pair probes covering both strands at six base pair spacing. A semi-quantitative estimate of transcript abundance for each gene could be obtained with this microarray design because the 5,419 *P. vivax* genes were probed by hundreds of independent oligonucleotides. While this array can be used to find noncoding RNAs, including antisense RNAs, the labeled cRNA for hybridization was prepared using a polyA reverse transcriptase priming method that would not give accurate descriptions of noncoding RNAs and thus we limited our analysis to predicted coding regions. In order to calculate an approximate gene expression level, *E*, we used the MOID algorithm, which ranks the probe intensity values for the 20 probes at the 3′ end of the transcript having similar GC values. It then assigns the expression value, *E*, to difference between the background (computed from thousands of probes with a similar GC content not predicted to be in the *P. vivax* or human genome) and the probe at the 70^th^ percentile (Table S1). Because the varying GC content of different *P. vivax* genes could give rise to spurious apparent expression levels, extensive optimization of probe selection was undertaken to ensure robust measurements of gene expression across all samples. We show that MOID expression values are not changed by selection of the independent 13^th^ or 14^th^ of 20 ranked probes (Methods S1 Figure IIIA). Additional optimization for different GC contents (Methods S1) shows that we can select probes from a whole-genome tiling array to accurately detect gene expression. To verify reproducibility of our analysis, we analyzed three samples, two asexual and one sporozoite in two technical replicates each, and obtained Pearson correlation coefficients of 0.986 to 0.996, indicating excellent reproducibility, whereas lower correlation is observed between samples from divergent asexual groups (Methods S1 Figure IIIB-D). Results were also confirmed by qRT-PCR, and compared to expressed sequence tags (ESTs), as described in Methods S1.

### Genes involved in similar processes are co-expressed

We first sought to address our hypothesis that genes involved in similar processes would be co-expressed. We identified 4,326 differentially expressed genes using the cutoff of ANOVA p-value <0.05 and fold change (FC) >2 using the individual probe intensity values for each gene using just our data. Differentially expressed genes were clustered using ontology-based pattern identification (OPI), an algorithm previously used with *P. falciparum* and *P. yoelii* expression datasets [10] (Figure 1A). This algorithm begins with 2,002 lists of genes sharing a common gene ontology (GO) annotation, literature co-citation, or other annotated parasite-specific process, e.g., there are 38 genes known to be involved in DNA replication. For each group a representative expression profile vector is computed using *E* values from all conditions for all genes in the group. Then all of the 4,326 differentially expressed genes are ranked by the correlation coefficient calculated between the gene's expression vector and the representative expression profile vector. The algorithm then uses a correlation coefficient optimization routine and creates expression clusters that contain the largest number of genes with common annotation and high correlation. In the case of the GO process “DNA replication” a group of 60 genes is created which contains 11 of 38 annotated DNA replication genes. The probability of this distribution occurring by chance is less than 10^−13^. Similarly, there is less than 10^−8^ probability of identifying 5 of 15 genes with an annotation of glycolysis in a cluster of 21 genes. In addition to using gene ontologies we also used other groupings of genes. In a previous analysis of *P. falciparum* lifecycle stages [9] we had identified 106 genes upregulated in sporozoites, which corresponds to 66 *P. vivax* orthologs in our dataset (GO:CCYCL01). Of these 35 were found in a group of 366 genes with a probability of enrichment by chance of 10^−20^. Overall these data showed that patterns of gene expression were nonrandom and that genes with similar functions showed much greater cohesion than would be expected by chance. Altogether, 121 clusters of highly correlated genes with shared annotation were identified (see companion web site: http://carrier.gnf.org/publications/Pv).

Previous analysis of *P. vivax* blood stage gene expression for parasites taken into synchronous short term culture has been performed [11] and we compared our results to these. Because this study involved two color microarrays and did not produce expression levels, direct comparisons between expression levels are not possible. However, we could still apply the same OPI clustering algorithm to the Bozdech data. Clustering of the Bozdech data (see companion web site) gave less information about sporozoites and sexual stages but revealed highly significant functional enrichments, especially within the area of protein biosynthesis and ribosome function, which is expected because of the higher sampling throughout the erythrocytic cycle. For example, 48 or the 58 annotated genes with a predicted role in cytosolic ribosome (GO:0022626) were found in a cluster of 126 genes, with a probability of enrichment by chances of 10^−63^. The data showed that in many cases the same genes that cluster with “cytosolic ribosome” in the Bozdech data also cluster with “small ribosomal subunit” in our data. The gene, PVX_084645, co-clusters with ribosomal genes in both cases and is listed as hypothetical but its *P. falciparum* ortholog, PF14_0360, is listed as an eukaryotic translation initiation factor 2A protein and thus its association with ribosomes is not surprising. PVX_101135, a hypothetical, clusters with ribosomal proteins in both cases. BLASTP (*p* = 1.3×10^−35^) shows a strong match to the yeast protein YOR091W, a protein of unknown function that associates with ribosomes that interacts with GTPase Rbg1p [20].

**Figure 2:**
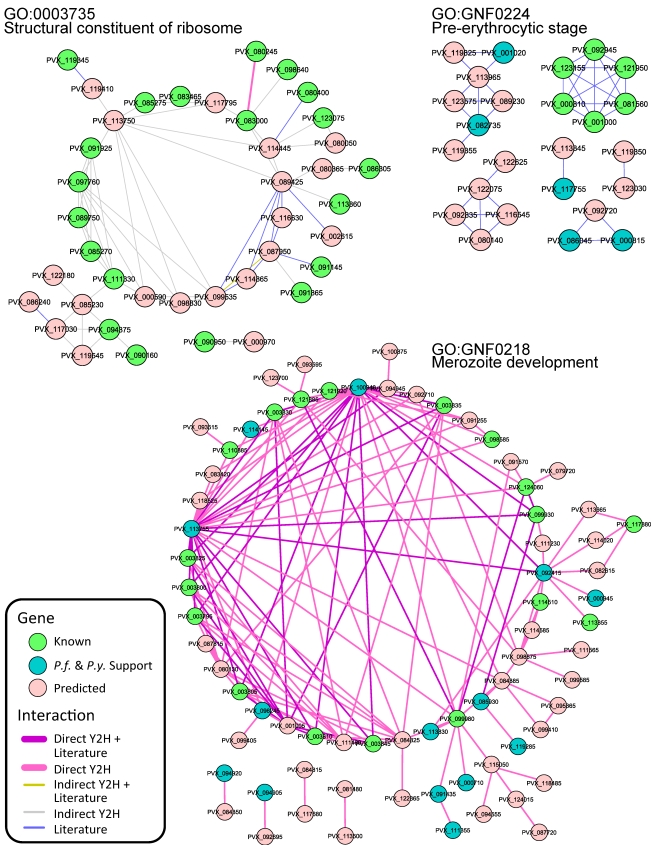
Network analysis. Corresponding *P. falciparum* orthologs of the cluster members may form statistically significant two-hybrid interaction networks. Gene members known to be involved in the cluster are colored in green, predicted ones are colored in pink and those supported by OPI analyses of *P. falciparum* and *P. yoelii* life cycle data set are shown in blue. Interaction edges are color coded to reflect whether it is a direct interaction or an indirect interaction, and whether it has literature co-citation support or not. A complete list of genes with cross-validation is given in Table S3.

We also co-clustered Bozdech data with our data to generate more accurate predictions of gene function creating a set of 192 different clusters containing between 2 and 493 genes and p-values between 10^−3^ and 10^−52^ (Figure 1, Table S2). Many of our functional predictions can be cross-validated with previously published data sets. In particular we checked if the same function prediction can be made based on combined *P. falciparum* and *P. yoelii* data set, using either previously published OPI clusters [10] or an updated cluster set using the latest gene annotations. For each *P. vivax* cluster we ran permutation tests to see their *P. falciparum* orthologs form denser protein networks than what would be expected by chance using both published two hybrid data [21] and literature co-citation data [10]. In total, 75 of the 192 OPI clusters led to protein networks with a p-value less than 0.05 based on 1000 permutation simulations. For example, PVX_123920, a putative ubiquitin-activating enzyme e1 clusters with genes involved in the proteosome regulatory particle in both *P. vivax* and in *P. falciparum* and has two-hybrid support as well [21]. While there are numerous examples that can be derived from well-studied processes, the greatest value of this data is in supporting predictions for genes that may not be found in other model organisms. PVX_092415 and PVX_113830 cluster with genes involved in merozoite development in *P. falciparum* and in *P. vivax* (GO:GNF0218) and furthermore, are supported by two-hybrid interaction studies from *P. falciparum* (Figure 2). Likewise, PVX_000945 shows a similar pattern. The *Toxoplasma gondii* homolog of this protein has been isolated from rhoptries [22] as has, the *Toxoplasma* ortholog of PVX_113800, which also clusters with genes involved in merozoite development in *P. falciparum*. There are numerous examples from pre-erythrocytic stages as well. Of course, some caution must be used in evaluating the data because genes involved in two different processes may be co-expressed (e.g. DNA replication is occurring during gamete production) and yet be involved in relatively different processes. Nevertheless this clustering exercise gives functional predictions for the many uncharacterized genes found in an OPI cluster.

### Heterogeneity in patient blood stage samples

**Figure 3:**
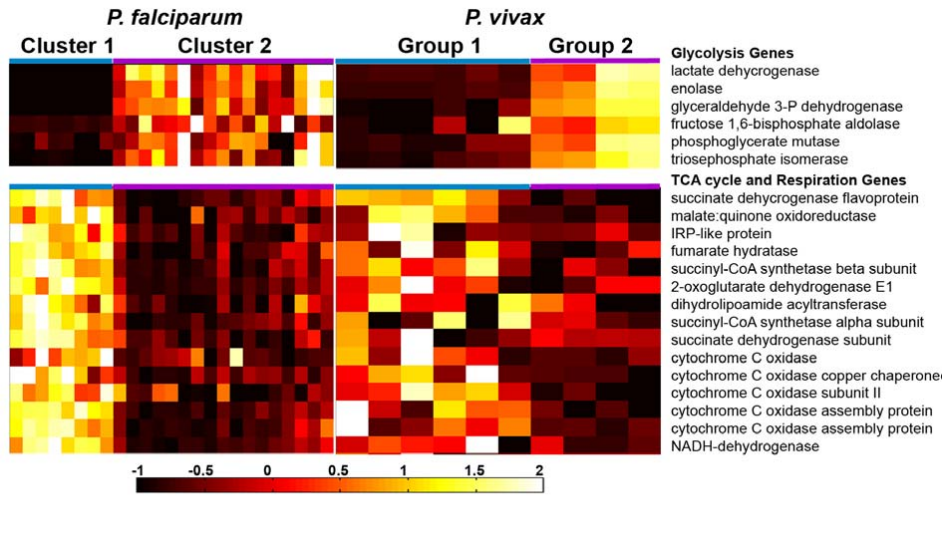
Expression of metabolic genes in *P. vivax* asexual blood stage parasites. The expression values of each gene in each sample were normalized by subtracting the average expression value and dividing by the standard deviation across all asexual samples. The glyceraldehyde 3-phosphate dehydrogenase gene is not annotated but the syntenic region in *P. vivax* also shows high expression in sample CM013 (Supplemental Figure 2). Gene expression values were normalized by subtracting the average expression value across all samples and dividing by the standard deviation of expression values across all samples. The resulting normalized expression values were colored on a scale ranging from −1 to +2, from black for the lowest, red for the middle, and white for the highest values. The gene ID numbers for glycolysis genes displayed in Figure 3 are: lactate dehydrogenase (PVX_116630, PF13_0141); enolase (PVX_095015, PF10_0155); glyceraldehyde 3-phosphate dehydrogenase (PVX_117321, PF14_0598); fructose 1,6-bisphosphate aldolase, putative (PVX_118255, PF14_0425); 2,3-bisphosphoglycerate-dependent phosphoglycerate mutase (PVX_091640, PF11_0208); triosephosphate isomerase (PVX_118495, PF14_0378). Gene ID numbers for TCA cycle and aerobic respiration genes are: flavoprotein subunit of succinate dehydrogenase (PVX_111005, PF10_0334); malate:quinone oxidoreductase (PVX_113980, PFF0815w); IRP-like protein (iron regulatory protein-like) (PVX_083005, PF13_0229); fumarate hydratase (PVX_099805, PFI1340w); ATP-specific succinyl-CoA synthetase beta subunit (PVX_084960, PF14_0295); 2-oxoglutarate dehydrogenase E1 component (PVX_089325, PF08_0045); dihydrolipoamide acyltransferase (PVX_119310, PFC0170c); succinyl-CoA synthetase alpha subunit (PVX_091100, PF11_0097); iron-sulfur subunit of succinate dehydrogenase (PVX_123345, PFL0630w); cytochrome C oxidase (PVX_099845, PFI1375w); cytochrome c oxidase copper chaperone (PVX_111430, PF10_0252); cytochrome c oxidase subunit II precursor (PVX_084995, PF14_0288); cytochrome c oxidase assembly protein (heme A: farnesyltransferase) (PVX_080280, PFE0970w); cytochrome c oxidase assembly protein (PVX_084785, PF14_0331); NADH dehydrogenase reaction protein (PVX_119700, PFC0505c).

Having established the overall quality of the data we examined the groups of genes that were differentially expressed in any one stage. While our initial expectation was that patient-derived blood samples would be relatively homogenous, unexpectedly, we observed large differences in the expression profiles of asexual samples with the most pronounced differences observed in genes involved in glycolysis. While we found little or no differential expression of the first three enzymes of the pathway: hexokinase (PVX_114315), glucose-6-phosphate isomerase (PVX_084735) and 6-phophofructokinase (PVX_099200), the eight remaining glycolytic enzymes: fructose 1,6-bisphosphate aldolase (PVX_118255), triosephosphate isomerase (PVX_118495), glyceraldehyde-3-phosphate dehydrogenase (PVX_117321), phosphoglycerate kinase (PVX_099535), phosphoglycerate mutase (PVX_091640), enolase (PVX_095015), lactate dehydrogenase (PVX_116630) and pyruvate kinase (PVX_114445) all showed very strong differential expression between samples with some genes showing up to a 100-fold higher expression in some asexual samples relative to others (Figure 3). These last eight enzymes are among the top 1% when ranked by transcript abundance *in vitro* trophozoites of *P. falciparum* and are among the top 5% of *P. vivax* genes in some samples but not others (Figure 3). These differences are unlikely to occur by chance (*p* = 1.9×10^−7^ comparing *P. vivax* group 1 and group 2 defined in Figure 3 with a paired t-test). While the high glycolysis samples were the minority (two of eight), they were more similar to that described in Cui *et al.* Here 22,236 ESTs from *P. vivax*-infected Thai blood samples [12] were sequenced. Our data showed good Spearman rank correlation with the Thai EST numbers and showed that those samples with high glycolysis gene *E* values were most similar (*r* = 0.53) (Table 1 and Methods S1) to the Thai strain. While it may be that these expression differences are due to contamination with gametocytes [23] the presence of 0–10% contaminating gametocytes cannot mathematically explain why lactate dehydrogenase is present at ∼3,300 units in two asexual samples and as low as 75 (almost indistinguishable from background) in others [24]. Furthermore, genes typically associated with gametocytogenesis (e.g. Pvs25, PVX_111175) are higher in the high glycolysis samples (CM12 and CM13). An alternative is that although morphologies looked similar, a substantially different proportion of early and late cell cycle stages were contained in high and low glycolysis samples. Genes typically associated with schizogony and invasion such as myosin motor proteins and reticulocyte binding proteins, transcribed later in the Bozdech erythrocytic cycle data, were expressed at higher levels in the low glycolysis samples (Figure 1). Despite this, glycolysis transcripts generally do not show 50–100 fold changes in expression levels throughout the *in vitro* erythrocytic cycle in *P. falciparum* cultured in vitro [8], [9] nor are such large fold changes observed with *P. vivax* cultured *in vitro* [11]. In addition, such a hypothesis would require the patient samples to have been tightly synchronized. While more samples will be need to be examined, the data raise an intriguing possibility that there may be differential regulation of metabolism in a subset of patient-derived samples as observed in *P. falciparum* patient–derived samples (Figure 3) [24].

### Many of the same transcripts needed for early intrahepatocyte function in *P. falciparum* are upregulated in *P. vivax* sporozoites

The molecular determinants regulating hypnozoite formation and relapse are unknown. One hypothesis is that the hypnozoite is an early stage exo-erythrocytic form (EEF) that is arrested in development. If this hypothesis is correct, we would expect the transcriptional profile of *P. vivax* sporozoites to be similar to species that do not form hypnozoites. Thus, we compared our *P. vivax* sporozoite expression profile to a *P. falciparum* sporozoite sample and three *P. yoelii* sporozoite samples analyzed previously [25], [26]. Sporozoite transcriptome comparisons showed that *P. vivax* sporozoites are generally similar to *P. yoelii* and *P. falciparum* sporozoites with positively correlated expression of highly expressed genes (*r* = 0.5). Despite some species-specific differences, we observed similar expression for many *P. vivax* genes whose *P. falciparum* and *P. yoelii* orthologs were previously shown to be upregulated in sporozoites, including some of the most highly expressed genes in the *P. vivax* sample (Table 2): In addition to genes known to be upregulated in sporozoites such as the initiation factor *UIS1*, and the serine threonine phosphatase *UIS2*, the list contains a number of known candidates for pre-erythrocytic subunit vaccines including the apical membrane antigen 1 (*AMA1*) gene and the circumsporozoite protein (*CSP*). In addition there are genes whose disruption has led to genetically attenuated sporozoites that may eventually be used in whole organism vaccines, including the ortholog of *P. falciparum* etramp10.3 or *UIS4* in *P. yoelii* [27], [28], *UIS3* [29] and *P52* [30]. From the set of genes upregulated in multiple species (Table S4) we identified a set of sporozoite conserved orthologous transcripts (SCOT) that were upregulated in multiple species but not yet annotated that may provide a list of possible candidates antigens for *P. vivax* pre-erythrocytic vaccines, or which when disrupted may yield genetically attenuated sporozoites.

**Table 2:** Most highly expressed *P. vivax* genes in the sporozoite stage.

Alias	Description	*P. vivax* Gene	Exp (E)	Rank
SCOT1	hypothetical protein, conserved in *Plasmodium*	PVX_122458*	17726	1
UIS4	UIS4, ETRAMP10.3	PVX_001715	16095	2
SCOT2	hypothetical protein, conserved	PVX_092505	13536	3
CSP1	circumsporozoite protein precursor, putative	PVX_119355	13189	4
PvSpz1	hypothetical protein	PVX_092255	8649	5
H3	histone H3, putative	PVX_114020	6864	6
SUI1	translation initiation factor SUI1, putative	PVX_101080	6656	7
PvSpz2	hypothetical protein, conserved	PVX_113796	6201	8
UVS1	hypothetical protein, conserved	PVX_089045	5424	9
AMA1	apical merozoite antigen 1	PVX_092275	4856	10
ECP1	cysteine protease serine-repeat antigen (SERA)	PVX_003790	4799	11
	Falstatin	PVX_099035	4358	13
UVS	hypothetical protein, conserved	PVX_122910	3975	14
PvROM1	rhomboid-like protease 1, putative	PVX_091350	3501	15
DIM1	DIM1 rRNA dimethylase, putative	PVX_100520	3383	17
	hypothetical protein, conserved	PVX_087095	3236	18
PvSpz3	hypothetical protein	PVX_114765	3074	19
S11	S11 *P. yoelii* conserved hypothetical protein	PVX_086200	3056	20
SCOT3	hypothetical protein, conserved	PVX_085040	2794	23
SCOT4	hypothetical protein, conserved	PVX_123360	2793	24
DHFR-TS	dihydrofolate reductase-thymidylate synthase	PVX_089950	2729	25
UVS2	protein tyrosine phosphatase, putative	PVX_091305	2713	26
UVS3	hypothetical protein, conserved	PVX_115405	2697	27
PvSpz4	hypothetical protein	PVX_118250	2688	28
SPECT	sporozoite microneme protein, putative	PVX_083025	2671	29
G10	G10 protein, putative	PVX_080110	2499	32
UIS2	UIS2, Ser/Thr protein phosphatase	PVX_117230	2332	34
D13	CCCH zinc finger domain D13 protein	PVX_089510	2282	35
UVS4	hypothetical protein, conserved	PVX_091250	2273	36
SCOT5	hypothetical protein, conserved	PVX_094795	2063	43
S14	S14 *P. yoelii* Sporozoite-specific gene	PVX_084410	2037	46
UVS5	hypothetical protein, conserved	PVX_122540	2022	47
PvSpz5	hypothetical protein, conserved in *Plasmodium*	PVX_091306*	1978	50

Selected highly expressed representative sporozoite genes. For complete details, see Table S1 and S3. Genes are ranked in descending order by maximum expression value in sporozoites. Sporozoite Conserved Orthologous Transcript (SCOT) genes are previously un-annotated or hypothetical genes upregulated in sporozoites of all *Plasmodium* species. UVS genes are upregulated only in *P. vivax* sporozoites, not in *P. falciparum* sporozoites. PvSpz genes are unique to *P. vivax*, with no orthologs in *P. falciparum*. * Indicates genes that were not previously annotated in *P. vivax*. PVX_122458 and PVX_091306 are new gene ID numbers according to their relative position in the genome and the gene models were based on the PKH_141170 and PKH_091040 gene models, respectively (Methods S1).

There are also, of course, genes which are not found in *P. falciparum* that are upregulated in *P. vivax* sporozoites and may play some unique role in the biology of *P. vivax* or other hypnozoite-forming parasites. Examples from the sporozoite specific combined OPI cluster (GO:GNF0006) include up to 63 genes without *P. falciparum* orthologs. There are also some genes previously shown to be upregulated in *P. falciparum* sporozoites, but whose orthologs were downregulated or barely increased in *P. vivax* sporozoites or vice versa (Table S4). One ApiAP2 gene (PVX_090110) and two zinc finger proteins (PVX_099045, PVX_099045, PVX_081725), which often function as transcription factors, were downregulated as were several RNA binding proteins (PVX_098995, PVX_098995, PVX_100715) and several kinases including two serine/threonine protein kinases (PVX_081395, PVX_081395, PVX_002805), a FIKK calcium-dependent protein kinase (PVX_091755) and a MAP kinase (PVX_084965). Although some of these differences could be due to strain specific or sporozoite collection variation we were able to confirm these species-specific differences in gene expression for 19 genes using qRT-PCR (Table S5).

### Genes upregulated in gamete/zygotes are mostly uncharacterized

The process of sexual development and meiosis are poorly characterized in many species. An expression profiles from the mix of macrogametes and zygotes (Table 1) derived from *in vitro* cultivation of fertilized patient-derived gametocytes showed upregulation of only a few characterized genes include those with roles in DNA replication, meiosis and chromatin structure (Table 3). The majority of strongly differentially expressed genes are uncharacterized, although many had been shown to be upregulated during induction of *in vitro* gametocytogenesis in *P. falciparum* [15]. As predicted, high expression of the *P. vivax* ortholog of Pvs25 (PVX_111175), the ookinete surface protein, is found in this stage. Another membrane protein gene, the ortholog of the *Toxoplasma gondii* PhIL1 (photosensitized INA-labeled protein 1) (PVX_081335), was among the most highly upregulated (>20-fold) in *P. vivax* gametes/zygotes. This protein associates with the cytoskeleton and is localized to the apical end of the plasma membrane [31] where it may function as an ookinete-specific surface protein for midgut invasion. The transcription factor high mobility group protein, HMGB2 (PVX_089520) is found among the top 20 genes expressed in this sample (See Table S1). It was shown to be a critical regulator of oocyst development and appears to activate genes that are most highly transcribed in gametocytes, but then stored and translated in ookinetes [32]. Interestingly, some of these uncharacterized proteins are also upregulated during meiosis in humans, such as the human orthologs of PVX_117890 [33]. An exceptionally large proportion of the genes upregulated in zygotes (Table 3) did not fall into clusters with enrichments of known genes, highlighting the problem of using “guilt by association” when the existing knowledge base is sparse.

**Table 3:** Genes showing the strongest probability of differential upregulation in zygotes.

Gene	Sexual stage ID	Description		Pf name	Panova	FC	Zygote E level
PVX_090155	Yes	tubulin alpha chain, putative		PFD1050w	5.78E-102	30.1	1434
PVX_090215	No	hypothetical protein		PFD1110w	2.29E-94	24.9	2588
PVX_111175	Yes	ookinete surface protein Pvs25		PF10_0303	2.05E-91	8.0	1882
PVX_101400	Yes	PFG377 protein		PFL2405c	2.40E-90	30.8	2352
PVX_096190	No	hypothetical protein		MAL7P1.124	1.03E-89	12.5	1677
PVX_114020	No	histone H3, putative		PFF0510w	3.78E-87	62.8	18297
PVX_089075	Yes	hypothetical protein		PF08_0024	4.18E-74	4.2	428
PVX_123425	No	hypothetical protein		PFL0715w	1.23E-71	10.6	1524
PVX_080425	Yes	transporter, putative		PFE0825w	1.51E-70	34.8	5350
PVX_118115	N.o.d.	hypothetical protein			5.83E-69	44.3	6918
PVX_094510	N.o.d.	hypothetical protein			1.44E-63	11.6	3533
PVX_123410	N.c.	hypothetical protein	PFL0700w		2.37E-60	7.7	933
PVX_117370	N.c.	hypothetical protein			2.94E-58	7.7	932
PVX_089570	Yes	meiotic recombination protein DMC1-like protein	MAL8P1.76		2.90E-57	24.4	3465
PVX_096300	N.c.	hypothetical protein	MAL7P1.107		2.41E-53	9.7	1636
PVX_085750	N.c.	hypothetical protein	PF14_0138		1.41E-51	5.2	521
PVX_099960	N.c.	hypothetical protein	PFI1465w		1.52E-51	12.01	3164
PVX_084615	No	DNA replication licensing factor MCM5, putative	PFL0580w		5.21E-51	3.0	328
PVX_081335	Yes	photosensitized INA-labeled protein 1	PFA0440w		1.43E-49	21.5	3918
PVX_089585	Yes	heat shock protein, putative	MAL8P1.78		1.46E-49	10.0	1903
PVX_092905	N.o.d.	hypothetical protein			1.51E-49	10.9	2081
PVX_082985	N.c.	hypothetical protein	MAL13P1.211		4.82E-48	8.4	1867
PVX_000675	N.c.	hypothetical protein	PFD0580c		8.01E-48	6.9	1301
PVX_117890	Yes	sortilin, putative	PF14_0493		4.72E-45	3.4	205
PVX_113490	N.o.d.	hypothetical protein	PFF0315c|		1.34E-44	14.8	2976

Sexual stage ID indicates that the *P. falciparum* or *P. yoelii* ortholog was part of a group upregulated previously shown to be upregulated during sexual development [10]. N.o.d. indicates that there was no orthologous data and N.c. indicates that orthologous data exists but that the gene was not found in clusters enriched for genes involved. Genes were ranked by p-value computed with the ANOVA method.

### Ookinete gene expression

Gene ontology-based OPI clustering revealed few annotated gene functions to be upregulated in ookinetes. This finding likely reflects the difficulty of obtaining sufficient quantities of this developmental stage for *in vitro* study. We found high expression of genes involved in chromatin (e.g. histones) and translation, potentially reflecting the fact that once the parasite reaches the midgut and forms an oocyst, thousands of rounds of DNA replication will likely commence. Genes specifically upregulated in oocysts were mostly uncharacterized. Ookinete surface protein genes such as the *P. vivax* ortholog of the CSP and TRAP-related protein (PVX_095475), previously shown to be essential for ookinete invasion [34], [35], the transmission-blocking target antigen Pfs230 (PVX_003905), a sexual stage antigen s48/45 domain containing protein (PVX_003900), the transmission blocking target antigen precursor Pfs48/45 (PVX_083235), showed modest levels of upregulation (<3-fold) in ookinetes relative to any other stage. In *P. falciparum*, transcripts for many of these peak in early stage gametocytogenesis [15], and thus high expression would not necessarily be expected in *P. vivax* ookinetes, even if protein is detected in present.

On the other hand there were a number of uncharacterized genes that showed substantial upregulation in ookinetes (>3-fold). These may encode the proteins that are needed for early oocyst formation. Some examples (see Table S1 for details) include a possible calcium dependent kinase, (PVX_083525), and 5-aminolevulinic acid synthase (PVX_101195), a key enzyme in the porphyrin synthesis pathway that leads to heme synthesis. Proteases and other degradative enzymes may be needed to exit the blood meal, penetrate the peritrophic matrix or form the oocyst on the mosquito midgut. A number of proteases were unregulated in ookinetes, including Ulp1 (ubiquitin-like protein-specific protease, PVX_100650), which is specifically required for cell cycle progression in other species.

### Drug resistance gene expression

While in many cases drug resistance in malaria parasites is conferred by single nucleotide polymorphisms [36]–[38], in some cases transcript amplification events in a target or in a pump [39] confer greater tolerance to drugs. We therefore compared levels of drug resistance gene transcripts to determine if anything interesting might be found. Most genes involved in drug resistance such as *pvcrt* (chloroquine resistance transporter, PVX_087980), *dhps* (dihydropteroate synthase, PVX_123230), *gtpch* (GTP cyclohydrolase, PVX_123830) or *mdr1* (multidrug resistance gene 1, PVX_080100) showed similar patterns in *P. vivax* and *P. falciparum*. An exception was dihydrofolate reductase (*dhfr*, PVX_089950), the target of the widely used antimalarial antifolate drug, pyrimethamine. Remarkably, *dhfr* expression was 50-fold higher in the India VII *P. vivax* sporozoites (confirmed by qRT-PCR, see Table S6) and 10-fold to 30-fold higher in *P. vivax* asexual stages compared to *P. falciparum*, where it was near background in all stages. Interestingly, while *P. falciparum* is sensitive to pyrimethamine, *P. vivax* is considered intrinsically resistant [40], although *in vitro* drug sensitivity data is not available for most *P. vivax* strains. Both *dhfr* mutations [41], [42] and amplifications [43] have been shown to confer resistance in *P. falciparum*. Thus, the higher *dhfr* expression in *P. vivax* relative to *P. falciparum* may confer higher tolerance to pyrimethamine.

### *vir* genes show lower than average expression

While much of our data shows that patterns of gene expression are conserved across *Plasmodium* species the data also reveals possible roles for information about the many uncharacterized genes which are specific to *P. vivax* including members of multigene families. The largest paralogous gene family of *P. vivax* is the diverse superfamily of variant surface protein genes (*vir*) found in subtelomeric regions [44] and may be related to the *rif* genes of *P. falciparum* and *yir* genes of *P. yoelii* [45]. The highly variable surface protein *var* gene family in *P. falciparum* has no orthologs in *P. vivax*. Expression levels for most of the 274 *vir* genes on the array were lower than for other genes. The average for *vir* genes was 210 units (approximately the 35^th^ percentile) using the highest value in any one stage (MaxExp in Supplemental Table 1), versus 572 units (approximately the 80^th^ percentile) on average for other genes. In addition 39 of the 109 genes in the genome, which were not detected as differentially expressed in any sample, were *vir* genes. Only 27 of the 274 probed *vir* genes showed strong differential regulation (>5-fold change, *p*_ANOVA_<0.001) versus 1,044 of the remaining 5,420 genes. Interestingly, four of the highest expressed *vir* genes (PVX_086860, PVX_096970, PVX_096980, PVX_096985) were among the highest expressed genes in the two high glycolysis asexual samples, indicating possible alternate mechanisms of expression control. Three of these highly expressed *vir* genes are adjacent to one another suggesting coordinated regulation of the entire region. Cui *et al.* also found these three *vir* genes and another gene in the same region (PVX_096975) had the highest numbers of ESTs in their patient samples [12].

### Most highly expressed members of multigene families are co-transcribed with exported proteins

Malaria parasites are known to be able to decorate the surface of infected erythrocytes with proteins that play roles in sequestration and immune evasion. Many of the exported proteins contain sequences, called PEXEL or VPS motifs that direct them out of the parasitophorous vacuole and to the surface [46], [47] and many are members of multigene families, presumably because enhanced levels of recombination between members or epigenetic transcriptional switching between members allows the parasite to evade the host immune responses to these exposed proteins. Many of the exported genes in *P. falciparum* are transcribed at specific, mid-trophozoite stages of the parasite cell cycle [47]. The number of predicted exported proteins in *P. vivax* is not as large as in *P. falciparum* most likely due to problems in recognizing PEXEL/VPS motifs in this species. For example, only 160 of the 346 *vir* proteins contain predicted export motifs [48]. In addition to *vir* genes the 20 members of the *P. vivax* PHIST (Plasmodia helical interspersed subtelomeric) exported gene family (Pv-fam-b)[49] on the array are expected to be exported as well as some members of the Pv-fam-h and Pv-fam-e families.

**Figure 4:**
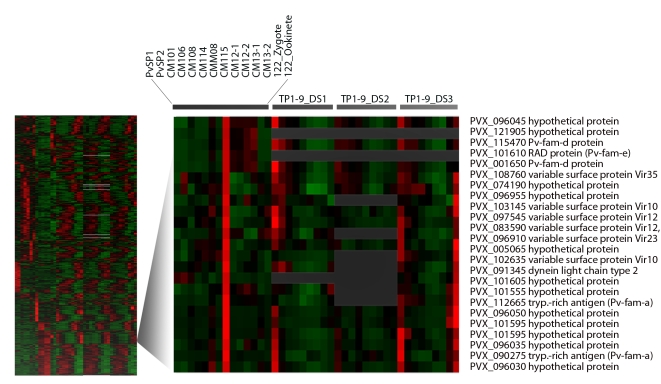
Co-expression of members of multigene families. Our entire dataset as well as that of Bozdech (TP samples) from three samples of parasites taken into short term culture were combined and subjected to hierarchical clustering. A portion of the dendrogram is expanded. In contrast to other areas of the dendrogram almost all genes are hypotheticals or members of multigene families, may of which are likely to be exported. Gray areas indicate experiments for which no data is available.

Remarkably many of the members of the multigene families as well as most predicted exported proteins that show strong differential transcription show peak expression in just one of our blood stage samples, CM115 (Figure 4). Five of the eight exported PHIST genes, six of the ten of Pv-fam-e family of RAD GTPases (some exported), nine of the 11, Pv-fam-d, and 9 or the 11 Pv-fam-a genes that show strong (>5X) differential expression again peak mostly in CM115. While *vir* genes show lower levels of expression overall, many of those that are differentially expressed also peak in CM115. Notably, 17 of the 38 expressed above 300 units show peak transcript levels in sample CM115. Many of the genes showing dramatic upregulation (up to 100 fold) in sample CM115 are abundantly transcribed. The Pv-fam-d family with 16 genes of unknown function has 2 genes in the top 1% of all genes ranked by maximum expression as does the Pv-fam-a family of tryptophan rich antigens (PvTRAg, average max expression in any one sample  = 1,969 units). One *P. vivax* tryptophan-rich antigen, PvTRAg (PVX_090265), has shown a very high seropositivity rate for the presence of antibodies in *P. vivax* malaria patients [50]. Another highly immunogenic antigen in this multigene family is PvATRAg74 (PVX_101510), recombinant versions of which showed erythrocyte binding activity and were recognized by all *P. vivax* patient sera tested [51]. This gene also ranks in the top 1% of transcripts in CM115. Many of the exported genes in *P. falciparum* are transcribed at specific, mid-trophozoite stages of the parasite cell cycle [47] and it seems likely that most of the parasites in asexual sample CM115 are at this export permissive stage. Thus many of the genes that are specifically upregulated in CM115 may play a role in immune evasion. While genes involved in DNA replication are also upregulated in CM115, these same genes are also upregulated in zygotes, while those encoding exported proteins may not be. These data nicely illustrate how the random collections of gene expression data that may be obtained from a neglected parasite can be used to create high quality predictions if enough random and yet diverse data is available. The data also illustrate that an advantage of using *P. vivax* patient samples is that a high level of synchrony may exist, that is confirmed by the Bozdech data (Figure 4).

Of course members of multigene families that share sequence similarity may have different cellular roles depending on their expression in the parasite lifecycle. An example is the group of cysteine protease genes referred to as serine repeat antigens (SERAs). In *P. falciparum*, PFB0325c, one of the seven SERA genes is expressed in sporozoites, while the others are expressed in blood stages. Disruption of this SERA ortholog, called *ECP1* in *P. berghei*, results in parasites that are unable to migrate out of the oocyst[52]. Although *P. vivax* contains 13 SERA cysteine protease paralogs, only the ECP1 ortholog (PVX_003790) was dramatically upregulated in sporozoites (105X). Two others showed substantial upregulation in sample CM12. None of the SERAs show maximal expression in sample CM115, nor do any members of the Pv-fam-c, homologous to the SURFIN gene family in *P. falciparum*. These are not appreciably expressed in any of our samples and may be functioning in other life stages.

### Exploring transcriptional regulatory circuits

In many organisms, including *Plasmodium* species, genes that are co-expressed share common sequence motifs in the regions upstream of their translational start sites [16]. To determine whether this would be true in *P. vivax* we looked for enriched motifs in various OPI groupings created with our data or the combined larger dataset. Here all possible 6–9 mers are counted within a group of co-expressed genes and these numbers are compared to the number found in all upstream regions. A corrected p-value that accounts for the multiple testing hypothesis is then computed. In general we find that transcriptional control is conserved and that many of the same motifs, which appear to control gene expression in *P. falciparum* also appear to control gene expression in *P. vivax*. For example an unbiased search for overrepresented sequences upstream of the 70 sporozoite-specific genes with promoter sequences (GO:GNF0006) relative to upstream regions in the whole genome found a motif similar to one associated with *P. falciparum* sporozoite genes. This putative regulatory sequence, TGCATG, is found upstream of 44 of the 70 *P. vivax* sporozoite-specific OPI cluster genes. The corrected probability of enrichment by chance in this set relative to the rest of the upstream regions in the genome is 0.02. Additionally, 48 of 65 genes in the list of SCOT genes contained the TGCATG motif (Table S6, *p* = 9.16×10^−7^). This is similar to the PfM24.1 sporozoite-specific regulatory motif CATGCAG identified in *P. falciparum* [16], sharing the core CATGC sequence and identical to the sequence (Table S6) bound by the *P. falciparum* ApiAP2 transcription factor PF14_0633 [53]. The *P. yoelii* ortholog of this transcription factor (PY00247) is among the highest expressed genes in midgut sporozoites [10], supporting the hypothesis that this protein functions as a specific activator of sporozoite transcription [25].

**Figure 5:**
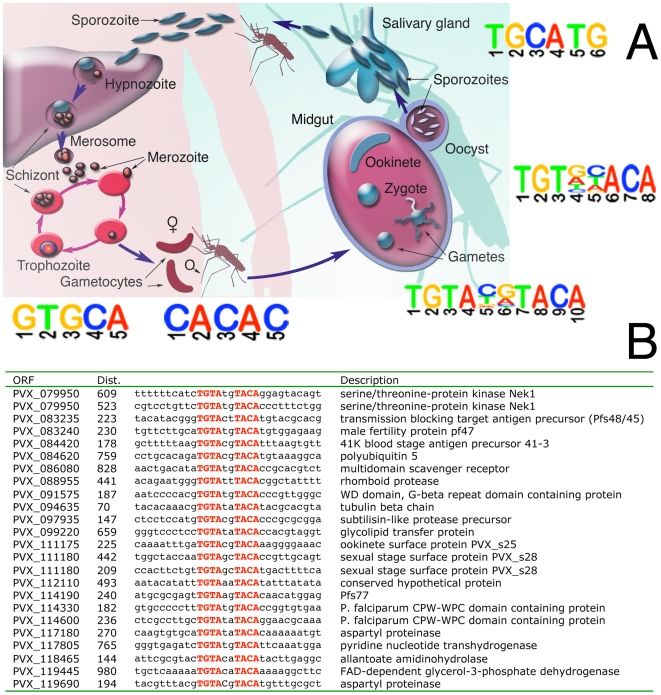
Sequence motifs associated with stage specific-expression. A. Figure showing different motifs associated with different lifecycle stage expression. B. Alignment of promoter regions showing the sexual development motif. The motif TGTAnnTACA was discovered in GO:PM16005087 in 92 of the 364 genes promoter regions. While the majority of the 92 genes were hypothetical, the 22 with a predicted function are shown here. Distance is the number of bases upstream of the translational start site.

A rich variety of other promoter motifs could be found by searching the various other OPI clusters as well. The sequence TGTAnnTACA was found enriched in the 1000 bases upstream of 383 genes from an OPI cluster containing 12 of the 19 genes mentioned in a an analysis of *P. falciparum* sexual development (GO:PM16005087, 92/364 genes, *p* = 1.69×10^−26^). This motif (Figure 5) is identical to the motif that is found in 65 of the 246 genes upregulated during sexual development in *P. falciparum* [15]. A cluster of genes enriched for ones with a role in gliding motility gave the motif TGTnnACA (86 or 155 genes, *p* = 0.00224). A search of a cluster with many genes predicted to be involved in merozoite development (GO:GNF0218) produced the motif GTGCA in 392 of 443 genes with a probability of enrichment by chance of 4.31×10^−13^. Protein binding microarrays have shown that the *P. falciparum* AP2 transcription factor, PFF0200c binds this motif [53]. It is also found upstream of *P. falciparum* genes transcribed during schizogony [16]. As with *P. falciparum* [54] the sequence CACAC was enriched in a cluster of genes containing an abundance of DNA replication genes (GO:0030894, 192 of 228 promoter regions, *p* = 4.7×10^−6^). Novel motifs were also found. A cluster enriched for genes identified in a male gametes (GO:PM16115694 [55]) yielded the motif CGTACA in 35 of 66 genes (*p* = 0.0645) and the sequence GCTATGC was found upstream of 36 of the 105 genes with promoter sequences in the cluster containing many of the structural constituents of the ribosome (GO:0003735, *p* = 0.007). This motif is similar to binding site for the AP2-O transcription factor (TAGCTA) that functions as a positive regulator of ookinete gene expression [56]. While these motifs may not be the same, expression of ribosomal proteins is upregulated in ookinete stages and one must keep in mind that transcription factor binding is likely to be combinatorial and a given gene may have multiple regulatory sites contained within its upstream region.

## Discussion

Analysis of the transcriptome of blood stages and sporozoites of *P. vivax* shows that the general mechanisms of growth, development, metabolism, and host-parasite interactions are shared by all *Plasmodium* species. Some of the expression differences that exist between species may provide insight into the molecular and genetic basis of biological differences that distinguish the species. The most significant differences include the formation of hypnozoites by *P. vivax*, pathogenic processes of sequestration and antigenic variation, and the wider geographical distribution of *P. vivax* into temperate regions. However, examination of a much larger number of different strains will probably be need to determine which differences of the differences we find are likely to be due to speciation and which are due to strain or experiment variability.

Because of the difficulties of working with *P. vivax*, transcriptional and proteomic studies represent one of the most effective ways to find candidate genes for vaccines or other processes. Many of the genes that cluster with proven antigens for pre-erythrocytic vaccines such as CSP may be worth investigating especially if sera drawn from individuals living in *P. vivax* endemic regions shows cross reactivity to their cognate protein [57]. A high-throughput analysis *P. falciparum* proteomic data [58] revealed one exceptionally abundant sporozoite protein named Ag2 in *P. falciparum* [59] or CelTos in *P. berghei* [60], which was more immunogenic than previously identified antigens such as CSP. In *P. falciparum* this is the third most abundant transcript in sporozoites. Ag2 one of the few antigens that is able to provide cross-species immunity and indeed we also find it highly expressed in our sporozoite sample. Given that many of the SCOT genes show strong expression only in sporozoite stages, their disruption may lead to genetically attenuated sporozoites that cannot develop in the liver, but which nevertheless provide immunity.

The gamete/zygote and ookinete expression data provides insight into mosquito midgut biology of a second human-infecting malaria parasite, and confirms common stage-specific gene expression shared by multiple *Plasmodium* species. Processes of sexual development, meiosis and DNA replication were evident in the gamete/zygote transcriptome. Some ookinete surface protein genes show highest expression in the gamete/zygote stage. Ookinetes produce stage-specific surface proteins, secreted proteases and invasion-related gliding motility proteins. While we observe upregulation of many cell-surface genes involved in invasion in our gametes/zygotes and ookinetes relative to asexual stages, this conclusion should be considered in light of the fact that our asexual stage gene expression represented ring and trophozoite stages, but no schizont and merozoite stage gene expression, thus biasing the analysis against the invasive blood stage parasites.

The function of the *vir* gene family is still unknown, but published data seems to indicate its function is fundamentally different from *var* genes. While *P. falciparum* var genes display mutually exclusive expression only during the mature stages [61], different *vir* [11], *yir* [62] and *rif* [63] genes are expressed in different intra-erythrocytic stages and gametocytes [64]. Previous studies showed that *vir* expression is not clonal, and multiple *vir* subfamilies are expressed in individual parasites from infected patients [65]. This is supported by our analysis of *vir* expression *in vivo*, with high-level expression of a small subset of *vir* genes in the high glycolysis samples along with other genes such as the PvTRAG genes that are likely involved in antigenic variation and immune evasion. However, examination of our data will also show that expression levels for many *vir* genes was very low or not detectable, indicated that they may be silenced. It is formally possibly that none of the parasites that we collected were at a stage where *vir* genes are being actively transcribed, potentially because of sequestration. The overall low levels could also be attributed to having mixed stage parasites in our samples, however many other cell-cycle regulated genes such as histones showed strong (>50) fold differential expression in some of our blood stage samples. Finally, *vir* gene genetic differences between the genome reference strain, Salvador I, and the Peruvian samples [66] could contribute to reduced expression levels, although high expression was certainly found for other members of variable gene families. Excepting these caveats, our data are consistent with a model in which some *vir* genes are transcriptionally-silenced.

The OPI analysis here provides functional predictions for a large numbers of genes. However, its limitation is that it uses existing knowledge and it is likely that interesting clusters of genes may be of mixed function, which can be estimated by examining the false positive and true positive rates in each cluster in Supplemental Table 2. In some cases the functional enrichment may be misleading and thus caution should be used in interpreting the labels. Many of the exported protein and blood stage antigens are found in an OPI cluster named “ribonuclease activity” reflecting the fact that this is the only well-annotated cellular process going on at this time. Finally, 501 of the differentially expressed genes were not contained in any of the OPI clusters. This may be because they are playing a role as ookinete, oocyst or hypnozoite function. Traditional hierarchical or *k*-means clustering is likely the best way to find out the function of these genes (see companion web site). Finally, 296 were not considered differentially expressed. Some of these may be upregulated in liver or oocyst sporozoite stages or they may be silenced.

One of the more remarkable things about the gene expression analysis is its robustness and insensitivity to sample contamination or admixture. Our group contained few samples focused on early sexual stage parasites and only a single zygote sample and yet we were able to extract groups of genes from which we could extract a sexual development transcription factor motif. Of course, the fact that we had no oocyst salivary gland sporozoite or liver stages means that this dataset is not comprehensive and more work will need to be done. While *P. vivax* is by no means a model organisms the gene expression data described here may be more useful for discovering motifs involved in regulating transcription as the lower AT content may be less problematic. It should also be noted that successes were not dependent on having the larger structured Bozdech dataset as many of the motifs could be extracted from the OPI clusters created with our data alone. A group of genes enriched for those with roles in sexual development (GO:PM16005087) could be extracted from our data when clustered independently (7 of the 19 genes in a group of 172, p = 10^−5^) and could be used in motif finding. However no groups of genes enriched for ones with roles in sexual development were found when the Bozdech data was analyzed independently (See Supplemental Table 2). However, when the datasets were combined, the quality of the cluster improved (12 of the 19 genes in a group of 246, p = 10^−8^) even though the Bozdech samples were not predicted to contain sexual stage parasites. Thus, more data and diversity is better.

These gene expression data for *in vivo* patient infections, gametes/zygotes, ookinetes and sporozoites of the *P. vivax* parasite provide an important foundation and reference for future studies. Numerous differences in gene expression suggest many hypotheses to be tested by researchers in the laboratory and in the field, and may be used to guide drug treatment and vaccine development for *P. vivax*. Further studies may provide correlations between *in vivo* parasite gene expression variability between patient samples and phenotypes of disease severity and drug resistance. In combination with accurate drug treatment outcomes and patient data, we will begin to identify the key determinants of the host-parasite interactions in this important pathogen.

## Supporting Information

Methods S1 Supplemental methods.
(0.81 MB DOC)

Table S1 Gene expression and ANOVA for all *P. vivax* genes.
(2.79 MB XLS)

Table S2 OPI clusters. Gene Ontology based OPI cluster summary and gene lists for Westenberger, Bozdech and merged datasets, each contained in different sheets.
(8.87 MB XLS)

Table S3 Annotation for newly predicted *P. vivax* genes.
(0.06 MB XLS)

Table S4 SCOT, UVS, DVS, and *P. vivax*-specific sporozoite gene lists and expression values.
(0.07 MB XLS)

Table S5 Quantitative RT-PCR values for differentially expressed sporozoite genes.
(0.04 MB XLS)

Table S6 Position and sequence of the putative regulatory motif in upstream regions of sporozoite-specific genes.
(0.07 MB XLS)
