# Supplementary material for: A Systems-Based Analysis of Plasmodium vivax Lifecycle Transcription from Human to Mosquito
Source: PLoS Negl Trop Dis. 2010 Apr 6;4(4):e653. doi: 10.1371/journal.pntd.0000653 (PMC2850316; doi:10.1371/journal.pntd.0000653)
Supplement: Methods S1 — Supplemental methods. (0.81 MB DOC) [file pntd.0000653.s001.doc]

**PLoS Neglected Tropical Diseases**

**Westenberger, et al. “**A systems-based analysis of *Plasmodium vivax* life-cycle transcription from human to mosquito.”

**Methods S1**

***P. vivax* tiling array probe selection.** All probes on the *P. vivax* tiling array were BLASTed against the coding sequences for the *P. vivax* genes and then we filtered out all probes that perfectly match with no mismatches to more than one place in the *P. vivax* genome. From the set of uniquely mapped probes we selected probes for evaluation of expression analysis. Because the array contains probes from both strands, we selected only probes that match to the CDS (sense). The mRNA is sense, the cRNA created from the Affymetrix RNA amplification is antisense. Therefore the sense probes detect the antisense cRNA.

Many overlapping probes on the *P. vivax* tiling array cover each gene. A probe selection algorithm was designed based on the following rules: A) probes with GC content closer to the optimal GC content of 9 is preferred based on comparison of signals between *P. vivax* probes and Affymetrix background probes; B) given probes of the same GC content, the probe closer to the 3' end is preferred; C) once a probe has been selected, its overlapping neighboring probes are deprioritized to minimize redundancy.

Let us use gene Pv090050 as an example for the purpose of illustration. Pv090050 consists of 31 probes with their GC counts ranging from 5 to 15, and 5'-distance ranging from 6 to 378. The selection algorithm scores all probes and the 30 best-scoring probes are shown in Supplemental Methods Figure I. The best 20 probes are used in later MOID calculation. The algorithm starts with 7 probes of GC count of 9 (Rule A) in the order of decreasing 5' distance (Rule B). Then it chooses 3 probe of GC count of 8 and 2 probes of GC count 10 (Rule A, B). The algorithm continues with probes of GC counts deviated further from 9. At the end, the best 20 non-overlapping probes has GC counts ranging from 7 to 11, with 5'-distance ranging from 6 to 378 covering most of the gene regions.

To validate the robustness of our probe selection algorithm against sources of bias or variability in GC content, we re-ran our algorithm using multiple different optimal GC contents and correlated the expression results from the different mechanism using Pearson Correlation. We found that using probes with GC=9 gave the most reproducible results with probes of similar GC contents from 8-10 GC per 25mer oligo probe (r>0.92). Whereas GC=12 or greater produced high variability (low Pearson correlations <0.6) that increased with increasing average GC content of the highly expressed genes in each sample (Supplemental Methods Table I). This is due to the nonlinear signal from higher GC content probes, resulting in much higher signal from more GC rich genes. Therefore, we decided to use the GC9 probe selection algorithm for all future expression analysis to minimize this potential bias. This is similar to our previous probe selection for *P. falciparum* expression arrays with average GC count of 7 in each 25mer. Additional information and analysis can be found on our companion web site http://carrier.gnf.org/publications/Pv.

**Background Subtraction**

For a probe of given GC content, the probability density function of its background noise *B*_GC_ is measured by all the background probes sharing the same GC content. That is, the probability of the background noise occurs at the level between *x* and *x*+*dx* is denoted by *B*_GC_(*x*)*dx*. Any observed probe intensity *E* is the sum of both true signal *E*_0_ and noise *E*-*E*_0_. Assuming all possible *E*_0_ values are equally likely, the expected value of true signal is:

*E*_0_ =$\int_{x=0}^{E} \left(E-x\right)B_{GC}\left(x\right)dx/\int_{x=0}^{E} B_{GC}\left(x\right)dx$.

For probe intensity much higher than the typical background signal, the above formula is equivalent to subtracting E by the mean of background signal, as found in many other background subtraction methods. However, for probe intensity closer to background intensity, the formula provides a probabilistic interpretation of the observation and guarantees a positively defined *E*_0_. The same background subtraction model has been previously validated[1,2].

**Expression level, standard deviation, and gene-specific noise**

For a given gene, signals from probes selected by the probe selection algorithm form an integral intensity distribution. According to the MOID algorithm, the 70 percentile of the intensity distribution is defined as the expression level of the gene[3]. For this study signal standard deviation (STDV) and noise level are defined based on bootstrapping approach. In an example bootstrapping run of Pv090050 consisting of 20 selected probes, signals from the 20 probes are sampled with replacement to form a new probe set (Figure II, red shaded region) and the MOID intensity is calculated. In this study such a bootstrapping calculation are repeated 25 times and results in a group of MOID intensities *S*, from which STDV is calculated. In a bootstrapping run of the noise calculation, each original signal probe is replaced by a randomly selected background probe of the same GC content, i.e., a virtual gene of the same GC distribution but only containing non-specific cross hybridization readings is constructed and the MOID intensity is calculated. In this study we construct 25 virtual genes for any given gene and obtain a group of noise estimation *N*. *S* and *N* are then log-transformed and a t-test is performed to obtain a p-value indicating the statistical significance of how the signal distribution deviating from the gene-specific background noise.

Normalization is performed as previously described[4]. First, genes with at least 6 probes and intensity value at least 1.5 above its noise estimation are retained. The normalization factor is determined so that the average for gene intensities between 30 and 90 percentile are scaled to 200. Quantile-based normalization algorithm was also tested, but not chosen because of its slightly inferior correlation within replicate samples.

Based on the RNA hybridization intensity, we identified genes that were expressed above background in each sample. To determine the criteria for calling a gene as “present” or expressed above background, we created sets of virtual control genes with a similar GC content to actual genes. We found that a signal to noise ratio >3.2 and a log_10_P value <-2 were the optimal cutoff criteria which excluded most virtual genes. This criteria resulted in between 4708 and 5347 of the 5417 total genes detected above background in each sample. (see companion web site).

As an additional test of the robustness of our MOID interpretation of the expression values, we performed correlations between expression values interpreted at the 65^th^, 70^th^, and 75^th^ percentile of probes. We found that the GC9 dataset that was chosen due to its high correlation with probe selection at GC8 and GC10, was also very highly correlated when different probe percentiles were used. Spearman Rank and Pearson correlations greater than 0.9 were found between 65^th^ and 70^th^ percentile for all samples. Slightly lower correlations were found for comparisons with 75^th^ percentile, which reflects the higher variability of GC rich probes, and the non-linear hybridization intensity of probes in the higher percentiles. Therefore, we believe that the use of the GC9 probe picking algorithm combined with MOID interpretation at the 70^th^ percentile is robust and reproducible with similar GC contents and probe percentiles, thus we used this interpretation of gene expression for all *P. vivax* RNA hybridizations.

**New P. vivax Gene Analysis**

We provide the putative gene coding region and amino acid sequence alignment of the hypothetical orthologs of PKH_141170, a SCOT gene highly expressed in sporozoites of *P. vivax* and *P. falciparum.* The gene is currently annotated in *P. knowlesi* and *P. chabaudi*, but all other genes are new predictions based on BLAST identity to the PKH_141170 gene.

>MAL13 | | 527065 to 527262 (reverse-complement)

ATGGAAACGATAATATCTCCAGTAATTACTTTACAACAAGCCCCTGTCGTTTATACAACG

ACATATAGAGTTGTACCACAAACAGTTGTATACACCTTTCCAAATAATATCCCTGTCGTT

AAAAATATACATGTGGTTCCTGCACAACAATTATGTCTTAGTTACGCCTATACTTCACCG

GTTACTGTAATAATATAA

>MALPY00640 | | 10172 to 10366 (reverse-complement)

ATGATTACAACAGTTGTATCACGATGGTTTACATTACAGTCAGCCCCAGTTGTTTACACA

ACTACATATAATGTAGTACCGCAAACGGTTGTTTATACTTTTCCTCAAAGTATACCGGTT

ATCAAAAACATTCAAGTATTCCGGCGCCACAGGTTTGTCTTAGCTATGCCTATACTTCTC

CTGTAACAGTAATAA

>PB_RP2745 | | 7929 to 8134 (reverse-complement)

ATGATTACAACAGTCGTATCACCGATGGTTACATTACAGTCAGCCCCAGTTGTTTACACA

ACTACATATAATGTAGTACCGCAAACGGTTGTCTATACTTTTCCTCAAAGCATACCAATT

ATCAAAAACATTCAAGTTATCCCGGCTCCCCAGGTTTGCCTTAGCTATGCCTATACTTCT

CCCGTAACAGTAATAATATAACATAA

>CM000455 | | 578725 to 578929 (reverse-complement)

ATGTTCGCAACAGTGATATCCCCCGTGGTGACGGTGCAGCCCGCGCCAGTTGTTTACACA

ACTACCTACAGTGTCGTGCCACAAACAGTTGTGTGCACGATTCCACAGACCATACCGATT

ATTAAAAATATTCAAGTTATCCCTTCCCAACAAGTATGTCTTAGCTACGCGTACGCCGCG

CCCGTAACGACTTTTATCCTTTAA

Clustal 2.0.10 multiple sequence alignment of PKH_141170 putative orthologs

PKH_141170 MFATVISPVVTVRPAPVVYTTTYSVVPQTVVYTIPQTIPIIKNIQVIPS 50

*P. vivax* MFATVISPVVTVQPAPVVYTTTYSVVPQTVVCTIPQTIPIIKNIQVIPS 50

*P. falciparum* METIISPVITLQQAPVVYTTTYRVVPQTVVYTFPNNIPVVKNIHVVPA 49

PC102342.00.0 MITTVVSPMVTLQSTPVVYTTTYNVVPQTVVYTFPQTIPIIKNIQVIPA 50

P. berghei MITTVVSPMVTLQSAPVVYTTTYNVVPQTVVYTFPQSIPIIKNIQVIPA 50

*P. yoelii* MITTVVSRWFTLQSAPVVYTTTYNVVPQTVVYTFPQSIPVIKNIQVFRR 48

: *::* .*:: :******** ******* *:*:.**::***:*.

PKH_141170 PQVCLSYSYAAPVTTVIL 67

*P. vivax* QQVCLSYAYAAPVTTFIL 67

*P. falciparum* QQLCLSYAYTSPVTVII- 65

PC102342.00.0 PQVCLSYAYTSPVTVII- 66

P. berghei PQVCLSYAYTSPVTVII- 66

*P. yoelii* HRFVL--AMETPILLL— 63

:. * : :*: .

We provide the putative DNA and protein sequence of the newly annotated gene Pv096306, and the alignment of its amino acid sequence with putative orthologs in other Plasmodium species.

Pv096306 Pv_PKH_031410_MAL7P1.105 2 exons of 86 and 258bp, similar to Pf with exons = 86,229 bp.

>CM000444 | | 608130 to 608645 (reverse-complement)

ATGCCGAACCATAAGACGTCCAGGGGCGAATGCTCCGACTACAACCGATCCAGGTGCTAC

AACCCGAAGGTGCATGTCTCCGGCTGG (splice)

TAAGAAGCACCCGCACGTGCGTGATTAGCTGCA

TCGCACTTTACATACACACATGTGCGTTGTGTATATATCCCTCCCAACTGTGTGTCCCCC

CCTTCCTTTTGCAACCAACCTCCCCCTAACACTCTCCTTTAAGAGAAAGGAACAAATAAG

CATTGCTCCCTCAAACCACCTTGCAGG (splice)

CACAACATTCAACACGATGAGGCCTACATACAA

AGCTACAACCGAATGCGTGAGTTCTACATGGAGGCGTACCCAACGGAGAGCATCAGCCAG

AAGTACCAGAGTGCCAGGGGGGGTGGGGCTCGAAAGAACCTGTCAGACAAGCGGGTGATT

TTCTACGAAGAGGGCGGGGAGGGCCACTGGGTCACCGAAAGCAGGCGCGCCTTTCGGGAG GGGGCGCGACGGAGCGGTGAAGTCCACCAAGCAGTGGCAGAGCGGTGA

>Pv096306 Pv_PKH_031410_MAL7P1.105 coding sequence (introns spliced out)

ATGCCGAACCATAAGACGTCCAGGGGCGAATGCTCCGACTACAACCGATCCAGGTGCTAC

AACCCGAAGGTGCATGTCTCCGGCTGGCACAACATTCAACACGATGAGGCCTACATACAA

AGCTACAACCGAATGCGTGAGTTCTACATGGAGGCGTACCCAACGGAGAGCATCAGCCAG

AAGTACCAGAGTGCCAGGGGGGGTGGGGCTCGAAAGAACCTGTCAGACAAGCGGGTGATT

TTCTACGAAGAGGGCGGGGAGGGCCACTGGGTCACCGAAAGCAGGCGCGCCTTTCGGGAG GGGGCGCGACGGAGCGGTGAAGTCCACCAAGCAGTGGCAGAGCGGTGA

>Pv096306 Pv_PKH_031410_MAL7P1.105 putative protein sequence

MPNHKTSRGECSDYNRSRCYNPKVHVSGW

HNIQHDEAYIQSYNRMREFYMEAYPTESISQKYQSARGGGARKNLSDKRVIFYEEGGEGHWVTESRRAFREGARRSGEVHQAVAER

CLUSTAL 2.0.10 multiple sequence alignment of MAL7P1.105 putative orthologs

PB000685.00.0 MSKENKFRDECLDYRRSQNYNPKVHVSGWYDIQNDEDYLEKYNKTKEFYIREYPSNPLEE 60

MAL7P1.105 MSNINTVKIDYSEYRTKRVYNPKVHVSGWYDIQNDLDYIEHYNKEKEFYIAEYPKNKFEK 60

Pv096306new MPNHKTSRGECSDYNRSRCYNPKVHVSGWHNIQHDEAYIQSYNRMREFYMEAYPTESISQ 60

PKH_031410 MSNHKMSRSEYSDYNRSRCYNPKVHVSGWYNIQHDDAYIQSYNRMRDFYMEAYPTERINQ 60

*.: : : : :*. .: **********::**:* *:: **: ::**: **.: :.:

PB000685.00.0 KYQNMS--KYRKNISDKEIIFYQD-HDTTYWETENKSSYKKNN------------- 100

MAL7P1.105 RYKNTNR-KSTKNISDKKVIFYQEGYESDSWLTENKESYKVDETK----------- 104

Pv096306new KYQSARGGGARKNLSDKRVIFYEEGGEGH-WVTESRRAFREGARRSGEVHQAVAER 115

PKH_031410 KYQSAKGDGARKNLSDKRVIFYEEGGACN-WITENRRAFKEEKRRSEDVCKAVTTE 115

:*:. **:***.:***:: * **.: :::

Sequencing full-length cDNAs by oligo-capping methods, Watanabe et al. found 11,262 sequences corresponding to 1566 *P. vivax* genes[5] expressed in asexual and gametocyte stages found in infected patient blood. Our current microarray analysis confirms expression of a majority of these cDNAs in blood samples. Almost all (98%) of these 1566 genes were found to be present above background levels in at least one of the blood samples, whereas 81% were expressed in either of the two sporozoite samples. The samples CMM12 and CMM13 in Group 2 had higher percentage (98%) expressed compared to the remaining blood samples representing Group 1 (94%).

**Comparison of E values to cDNA sequencing.** An advantage of microarrays with multiple probes per gene is that through analysis of the distribution of probe intensities for each gene an estimate of transcript abundance can be obtained, which is comparable to read number for EST sequencing, given sufficient depth of sequencing to provide accurate quantitation of all transcripts. For example, Cui et al generated 22,236 EST sequences from Thai blood samples that contained a mixture of asexual and gametocyte forms[6]. We found good concordance between our data and this EST dataset. Since the *P. vivax* genome was not sequenced at the time of this publication, ESTs were previously assigned to GenBank sequences. We have re-analyzed these sequences by performing a BLAST search of all ESTs against all *P. vivax* annotated transcripts. We identified the gene represented by the EST as the top-scoring hit with a match greater than 50bp long. We identified 3543 genes with at least one EST, 2508 with at least two ESTs, 1110 with at least 5 ESTs and 463 with at least 10 ESTs. The numbers of ESTs for genes with fewer ESTs may not accurately reflect the gene expression due to the stochastic sampling of ESTs by shotgun sequencing. Therefore, we performed Pearson and Spearman rank correlations between the Cui, et al. data and our expression data for asexual blood stages.

Of the top 25 ESTs, four could not be linked to any current gene annotation, but 17 of the remaining 20 with 6 or more probes on the array (note that data for genes with fewer than 6 probes should not be included in this calculation) were found in the top 5% of genes expressed in asexual blood samples (See Supplemental Table I). One exception was the gene annotated as encoding a senescence protein (PVX_088865) that had the highest number of ESTs assigned to it from the Thai EST dataset, but which showed only moderate expression in our Peruvian samples. This gene is adjacent to an un-annotated ribosomal RNA in *P. falciparum* on its 3’ end and BLAST analysis of a highly transcribed region from the 5’ end shows identity to the 35S RNA transcript. A ribosomal RNA gene has recently been annotated in this syntenic region adjacent to PVX_088865. Because we find that ribosomal RNA transcripts are often very abundant, even in poly-A primed cDNA preparations it is likely that the adjacent rRNA promoter contributed to the large number of ESTs assigned to this gene in the Thai samples. Interestingly, the EST that ranks 4^th^ in the EST project matches *P. falciparum* glyceraldehyde 3-phosphate dehydrogenase. The *P. vivax* ortholog was not annotated in the PlasmoDB version 5.4 and thus we looked for evidence that it would be found in the region syntenic to the *P. falciparum* ortholog. Indeed, we found a very highly transcribed region showing the predicted exon structure in the syntenic location on contig 7179 (Supplemental Methods Figure IV).

**References**

1. Zhou Y, Ramachandran V, Kumar KA, Westenberger S, Refour P, et al. (2008) Evidence-Based Annotation of the Malaria Parasite's Genome Using Comparative Expression Profiling. PLoS ONE 3: e1570.

2. Kidgell C, Volkman SK, Daily J, Borevitz JO, Plouffe D, et al. (2006) A systematic map of genetic variation in Plasmodium falciparum. PLoS Pathog 2: e57.

3. Zhou Y, Abagyan R (2002) Match-only integral distribution (MOID) algorithm for high-density oligonucleotide array analysis. BMC Bioinformatics 3: 3.

4. Le Roch KG, Zhou Y, Blair PL, Grainger M, Moch JK, et al. (2003) Discovery of gene function by expression profiling of the malaria parasite life cycle. Science 301: 1503-1508.

5. Watanabe J, Wakaguri H, Sasaki M, Suzuki Y, Sugano S (2007) Comparasite: a database for comparative study of transcriptomes of parasites defined by full-length cDNAs. Nucleic Acids Res 35: D431-438.

6. Cui L, Fan Q, Hu Y, Karamycheva SA, Quackenbush J, et al. (2005) Gene discovery in Plasmodium vivax through sequencing of ESTs from mixed blood stages. Mol Biochem Parasitol 144: 1-9.

| PVDP08042501: PvSPZ1 | | | |  |  | PVDP08040201: CMM08 | | | |  |  |
| --- | --- | --- | --- | --- | --- | --- | --- | --- | --- | --- | --- |
| Method | GC8 | GC9 | GC10 | GC11 | GC12 | Method | GC8 | GC9 | GC10 | GC11 | GC12 |
| GC8 | 1.00 | 0.97 | 0.92 | 0.90 | 0.84 | GC8 | 1.00 | 0.96 | 0.93 | 0.88 | 0.75 |
| GC9 | 0.97 | 1.00 | 0.94 | 0.92 | 0.85 | GC9 | 0.96 | 1.00 | 0.94 | 0.89 | 0.77 |
| GC10 | 0.92 | 0.94 | 1.00 | 0.94 | 0.89 | GC10 | 0.93 | 0.94 | 1.00 | 0.91 | 0.79 |
| GC11 | 0.90 | 0.92 | 0.94 | 1.00 | 0.92 | GC11 | 0.88 | 0.89 | 0.91 | 1.00 | 0.82 |
| GC12 | 0.84 | 0.85 | 0.89 | 0.92 | 1.00 | GC12 | 0.75 | 0.77 | 0.79 | 0.82 | 1.00 |
| PVDP08042502: PvSPZ2 | | | |  |  | PVDP08042507: CM115 | | | |  |  |
| Method | GC8 | GC9 | GC10 | GC11 | GC12 | Method | GC8 | GC9 | GC10 | GC11 | GC12 |
| GC8 | 1.00 | 0.97 | 0.94 | 0.91 | 0.82 | GC8 | 1.00 | 0.95 | 0.90 | 0.84 | 0.77 |
| GC9 | 0.97 | 1.00 | 0.96 | 0.93 | 0.86 | GC9 | 0.95 | 1.00 | 0.94 | 0.89 | 0.81 |
| GC10 | 0.94 | 0.96 | 1.00 | 0.95 | 0.89 | GC10 | 0.90 | 0.94 | 1.00 | 0.93 | 0.85 |
| GC11 | 0.91 | 0.93 | 0.95 | 1.00 | 0.90 | GC11 | 0.84 | 0.89 | 0.93 | 1.00 | 0.89 |
| GC12 | 0.82 | 0.86 | 0.89 | 0.90 | 1.00 | GC12 | 0.77 | 0.81 | 0.85 | 0.89 | 1.00 |
| PVDP08042503: CM101 | | | |  |  | PVDP08042508: CM12-1 | | | |  |  |
| Method | GC8 | GC9 | GC10 | GC11 | GC12 | Method | GC8 | GC9 | GC10 | GC11 | GC12 |
| GC8 | 1.00 | 0.96 | 0.90 | 0.80 | 0.58 | GC8 | 1.00 | 0.97 | 0.94 | 0.91 | 0.89 |
| GC9 | 0.96 | 1.00 | 0.92 | 0.81 | 0.60 | GC9 | 0.97 | 1.00 | 0.97 | 0.95 | 0.92 |
| GC10 | 0.90 | 0.92 | 1.00 | 0.83 | 0.62 | GC10 | 0.94 | 0.97 | 1.00 | 0.96 | 0.93 |
| GC11 | 0.80 | 0.81 | 0.83 | 1.00 | 0.71 | GC11 | 0.91 | 0.95 | 0.96 | 1.00 | 0.95 |
| GC12 | 0.58 | 0.60 | 0.62 | 0.71 | 1.00 | GC12 | 0.89 | 0.92 | 0.93 | 0.95 | 1.00 |
| PVDP08042504: CM106 | | | |  |  | PVDP08042509: CM12-2 | | | |  |  |
| Method | GC8 | GC9 | GC10 | GC11 | GC12 | Method | GC8 | GC9 | GC10 | GC11 | GC12 |
| GC8 | 1.00 | 0.96 | 0.91 | 0.74 | 0.59 | GC8 | 1.00 | 0.97 | 0.93 | 0.91 | 0.89 |
| GC9 | 0.96 | 1.00 | 0.92 | 0.76 | 0.61 | GC9 | 0.97 | 1.00 | 0.97 | 0.95 | 0.93 |
| GC10 | 0.91 | 0.92 | 1.00 | 0.78 | 0.62 | GC10 | 0.93 | 0.97 | 1.00 | 0.97 | 0.95 |
| GC11 | 0.74 | 0.76 | 0.78 | 1.00 | 0.69 | GC11 | 0.91 | 0.95 | 0.97 | 1.00 | 0.96 |
| GC12 | 0.59 | 0.61 | 0.62 | 0.69 | 1.00 | GC12 | 0.89 | 0.93 | 0.95 | 0.96 | 1.00 |
| PVDP08042505: CM108 | | | |  |  | PVDP08042510: CM13-1 | | | |  |  |
| Method | GC8 | GC9 | GC10 | GC11 | GC12 | Method | GC8 | GC9 | GC10 | GC11 | GC12 |
| GC8 | 1.00 | 0.95 | 0.89 | 0.71 | 0.48 | GC8 | 1.00 | 0.97 | 0.92 | 0.88 | 0.88 |
| GC9 | 0.95 | 1.00 | 0.91 | 0.73 | 0.51 | GC9 | 0.97 | 1.00 | 0.96 | 0.94 | 0.93 |
| GC10 | 0.89 | 0.91 | 1.00 | 0.76 | 0.53 | GC10 | 0.92 | 0.96 | 1.00 | 0.96 | 0.95 |
| GC11 | 0.71 | 0.73 | 0.76 | 1.00 | 0.63 | GC11 | 0.88 | 0.94 | 0.96 | 1.00 | 0.97 |
| GC12 | 0.48 | 0.51 | 0.53 | 0.63 | 1.00 | GC12 | 0.88 | 0.93 | 0.95 | 0.97 | 1.00 |
| PVDP08042506: CM114 | | | |  |  | PVDP08042511: CM13-2 | | | |  |  |
| Method | GC8 | GC9 | GC10 | GC11 | GC12 | Method | GC8 | GC9 | GC10 | GC11 | GC12 |
| GC8 | 1.00 | 0.98 | 0.93 | 0.75 | 0.52 | GC8 | 1.00 | 0.97 | 0.92 | 0.88 | 0.88 |
| GC9 | 0.98 | 1.00 | 0.94 | 0.77 | 0.54 | GC9 | 0.97 | 1.00 | 0.96 | 0.93 | 0.92 |
| GC10 | 0.93 | 0.94 | 1.00 | 0.79 | 0.57 | GC10 | 0.92 | 0.96 | 1.00 | 0.96 | 0.95 |
| GC11 | 0.75 | 0.77 | 0.79 | 1.00 | 0.67 | GC11 | 0.88 | 0.93 | 0.96 | 1.00 | 0.96 |
| GC12 | 0.52 | 0.54 | 0.57 | 0.67 | 1.00 | GC12 | 0.88 | 0.92 | 0.95 | 0.96 | 1.00 |

Supplemental Methods Table I. Pearson Correlation Coefficients of data from different probe selection criteria. The P. vivax has an isochore structure with some classes of genes having different GC contents. To validate the robustness of our probe selection algorithm against sources of bias or variability in GC content, we re-ran our algorithm using multiple different optimal GC contents and correlated the expression results from the different mechanism using Pearson Correlation. We found that using probes with GC=9 gave the most reproducible results with probes of similar GC contents from 8-10 GC per 25mer oligo probe (r>0.92). Whereas GC=12 or greater produced high variability (low Pearson correlations <0.6) that increased with increasing average GC content of the highly expressed genes in each sample. This is due to the nonlinear signal from higher GC content probes, resulting in much higher signal from more GC rich genes. Therefore, we decided to use the GC9 probe selection algorithm for all future expression analysis to minimize this potential bias. This is similar to our previous probe selection for *P. falciparum* expression arrays with average GC count of 7 in each 25mer.

| Description | *P. vivax* Gene | *P. falciparum* Ortholog | Group 1 Avg Exp (E) | Group 2 Avg Exp (E) | Cui et al. ESTs |
| --- | --- | --- | --- | --- | --- |
| histone H2B | PVX_090935 | PF11_0062 | 3699 | 19835 | 91 |
| early transcribed membrane protein 10.1 | PVX_003565 | PFL1945c | 3793 | 16659 | 313 |
| histone H4, putative | PVX_090930 | PF11_0061 | 1972 | 12723 | 30 |
| early transcribed membrane protein 4 | PVX_090230 | PFD1120c | 1743 | 11474 | 344 |
| glyceraldehyde-3-phosphate dehydrogenase | PVX_117321 | PF14_0598 | 1027 | 11140 | 375 |
| Pv-fam-d protein | PVX_113235 |  | 1366 | 8590 | 18 |
| histone H2A, putative | PVX_114015 | PFF0860c | 491 | 8396 | 15 |
| histone H3, putative | PVX_114020 | PFF0865w | 1812 | 7497 | 25 |
| hypothetical protein, conserved | PVX_113796 | PFF0640w | 12029 | 6299 | 2 |
| 40S ribosomal protein S20e, putative | PVX_094375 | PF10_0038 | 727 | 6062 | 35 |
| tryptophan-rich antigen (Pv-fam-a) | PVX_112690 |  | 396 | 5963 | 32 |
| ribosomal protein L3, putative | PVX_111330 | PF10_0272 | 785 | 5711 | 3 |
| hypothetical protein, conserved | PVX_101490 | PFL2515c | 1771 | 5665 | 37 |
| Hypothetical protein, conserved in *Plasmodium* | PVX_097581 |  | 1424 | 5660 | 0 |
| translation initiation factor SUI1, putative | PVX_101080 | PFL2095w | 1230 | 5378 | 35 |
| enolase, putative | PVX_095015 | PF10_0155 | 254 | 5169 | 60 |
| glutaredoxin, putative | PVX_119440 | PFC0271c | 404 | 5076 | 61 |
| hypothetical protein, conserved | PVX_123351 | PFL0637c | 313 | 4641 | 0 |
| fructose 1,6-bisphosphate aldolase, putative | PVX_118255 | PF14_0425 | 791 | 4622 | 39 |
| elongation factor 1 alpha, putative | PVX_114830 | PF13_0305 | 430 | 4307 | 84 |
| hypothetical protein | PVX_121935 |  | 1066 | 4178 | 88 |
| 40S ribosomal protein S4, putative | PVX_090950 | PF11_0065 | 308 | 4064 | 47 |
| 60S ribosomal protein L41, putative | PVX_092820 |  | 1601 | 3787 | 8 |
| 40S ribosomal protein S5, putative | PVX_096265 | PF07_0088 | 807 | 3599 | 21 |
| 40S ribosomal protein S27, putative | PVX_122245 | PF13_0045 | 1203 | 3483 | 44 |
| Pv-fam-d protein | PVX_101520 |  | 433 | 3474 | 34 |
| 40S ribosomal protein S21, putative | PVX_092805 | PF11_0454 | 1060 | 3434 | 134 |
| hypothetical protein, conserved | PVX_123060 | PF08_0074 | 205 | 3392 | 35 |
| transporter, putative | PVX_080425 | PFE0825w | 541 | 3389 | 14 |
| circumsporozoite-protein related antigen | PVX_091700 | PF11_0224 | 369 | 3351 | 65 |
| sec61 beta 1 subunit, putative | PVX_089275 | MAL8P1.51 | 970 | 3314 | 30 |
| hypoxanthine phosphoribosyltransferase | PVX_094840 | PF10_0121 | 181 | 3304 | 55 |

Supplemental Methods Table II. Most highly expressed genes in asexual samples. EST sequences generated by Cui *et al.* [6] were mapped to *P. vivax* genes by BLAST. Genes are ranked by their highest expression in Group 2, a high glycolysis sample (See Figure III for definition), which is most similar to the EST results, and to *in vitro* gene expression. Avg Exp is the average expression of all samples in each group.


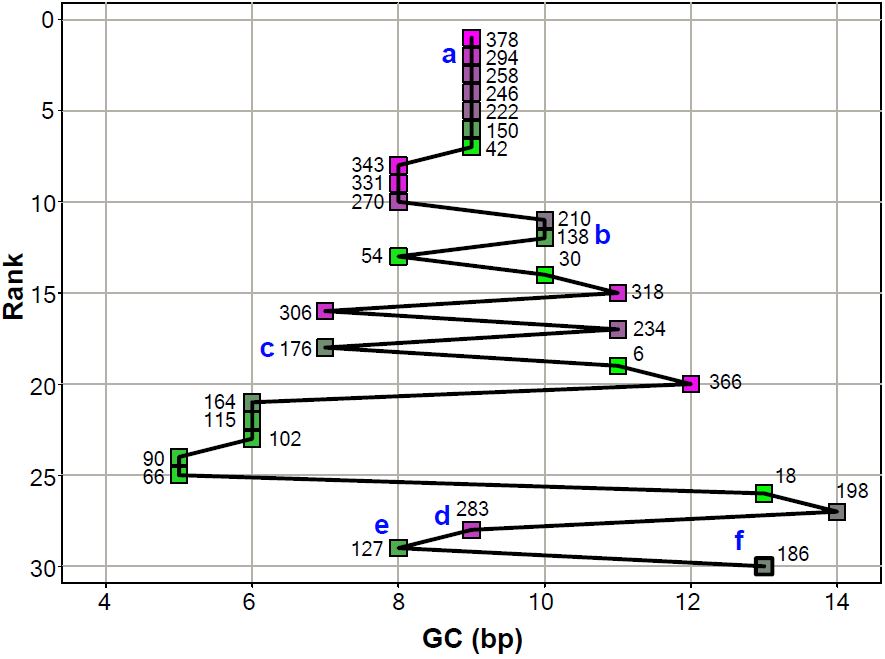


Supplemental Methods Figure I. Example of the probe selection algorithm. The ranking of probes for Pv090050. Each probe is represented by a solid rectangle color by its 5'-distance, of which the numeric value is also shown as its label. Probes d, e, and f are deprioritized due to their close distance with probes a, b, and c, respectively.


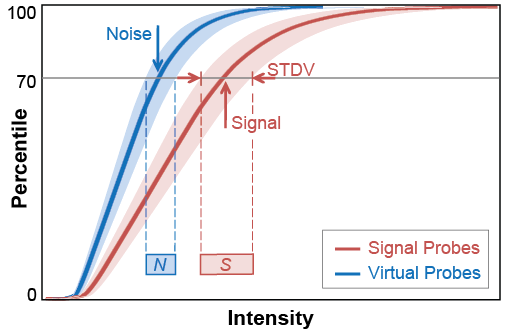


Supplemental Methods Figure II. Schematic representation of MOID gene expression algorithm.


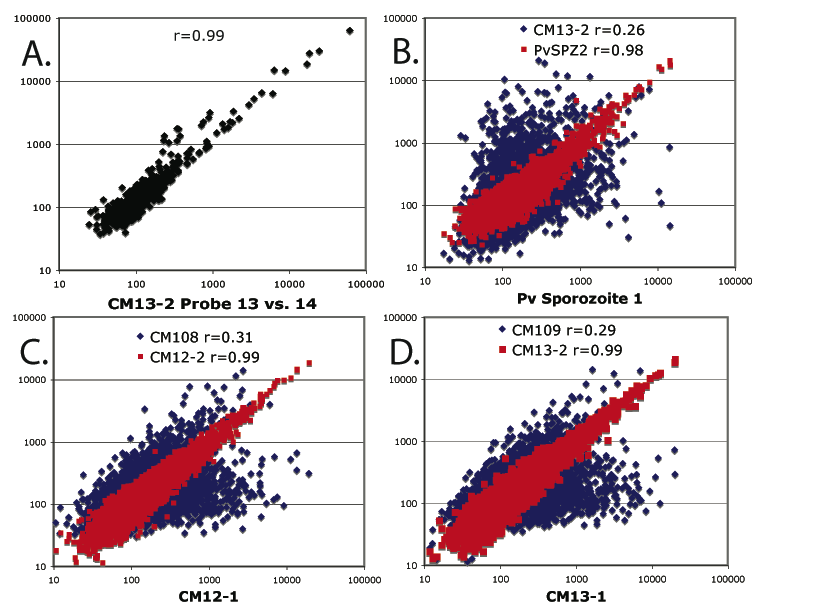


Supplemental Methods Figure III. Scatterplot comparison of *P. vivax* expression data.

MOID-normalized gene expression values for all 5,417 genes were plotted on a log scale. A) Comparison of CM13-2 sample expression values derived using different probes for the same gene. Using a set of 20 different 25mer probes for each gene, the MOID algorithm derives a semi-quantitative expression level, E, from the intensity value of 25mer probe whose intensity ranks at the 70th percentile.  Comparison of CM13-2 sample E values obtained using the probe at the 65th versus 70th percentile show that the calculated E value is relatively insensitive to probe choice, providing highly robust and reproducible gene expression interpretation from independent probe selection.  B) Technical replicates of the *P. vivax* sporozoite sample are highly correlated, with low correlation to asexual Group 2 sample CM13-2. C) Technical replicates of asexual Group 2 sample CM012 are highly correlated to each other compared to asexual Group 1 sample CM108. D) Technical replicates of asexual Group 2 sample CM13 are highly correlated compared to asexual Group 1 sample CM109.


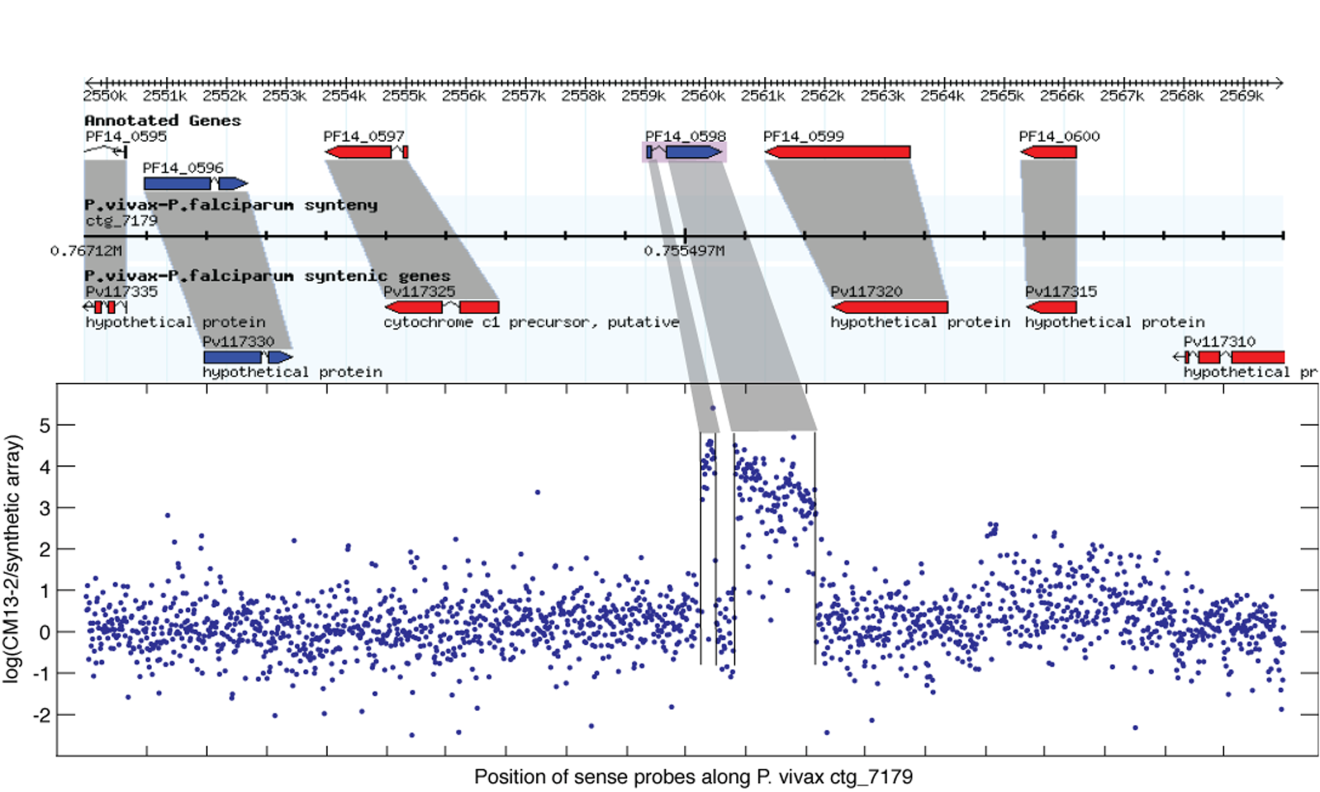


Supplemental Methods Figure IV. Transcriptional evidence for glyceraldehyde 3-phosphate dehydrogenase.

The gene for glyceraldehyde 3-phosphate dehydrogenase is not annotated in PlasmoDB, but we provide the putative gene number Pv117321. Chromosome synteny alignment between *P. falciparum* and *P. vivax* was taken from PlasmoDB Genome Browser. The probe intensities across all *P. vivax* expression datasets were averaged to create a synthetic baseline array in the absence of a genomic DNA hybridization. The log ratios of probe intensities for sample CM013-2 versus the synthetic array clearly demonstrate the high expression of the glyceraldehyde 3-phosphate dehydrogenase gene in the expected syntenic region with identical exon structure to the *P. falciparum* ortholog. Only sense probes are displayed. The lines were drawn by visual inspection.
